# Improved Calcium Homeostasis and Force by Selenium Treatment and Training in Aged Mouse Skeletal Muscle

**DOI:** 10.1038/s41598-020-58500-x

**Published:** 2020-02-03

**Authors:** János Fodor, Dána Al-Gaadi, Tamás Czirják, Tamás Oláh, Beatrix Dienes, László Csernoch, Péter Szentesi

**Affiliations:** 1 Department of Physiology, Faculty of Medicine, University of Debrecen, Debrecen, Hungary; 2 Doctoral School of Molecular Medicine, University of Debrecen, Debrecen, Hungary

**Keywords:** Ageing, Calcium and vitamin D

## Abstract

During aging reduction in muscle mass (sarcopenia) and decrease in physical activity lead to partial loss of muscle force and increased fatigability. Deficiency in the essential trace element selenium might augment these symptoms as it can cause muscle pain, fatigue, and proximal weakness. Average voluntary daily running, maximal twitch and tetanic force, and calcium release from the sarcoplasmic reticulum (SR) decreased while reactive oxygen species (ROS) production associated with tetanic contractions increased in aged – 22-month-old – as compared to young – 4-month-old – mice. These changes were accompanied by a decline in the ryanodine receptor type 1 (RyR1) and Selenoprotein N content and the increased amount of a degraded RyR1. Both lifelong training and selenium supplementation, but not the presence of an increased muscle mass at young age, were able to compensate for the reduction in muscle force and SR calcium release with age. Selenium supplementation was also able to significantly enhance the Selenoprotein N levels in aged mice. Our results describe, for the first time, the beneficial effects of selenium supplementation on calcium release from the SR and muscle force in old age while point out that increased muscle mass does not improve physical performance with aging.

## Introduction

Muscle mass continuously decreases with age, a process which accelerates in the seventh decade and beyond^1^, while the biological processes involved in these mechanisms are not fully understood. The repair capacity and turnover of muscles also reduce with age. A potential candidate could be the decreased basal metabolic rate which contributes to the drop in lean body muscle mass. As such, the phenomena would lead to reduced physical activity, nutritional deficits, chronic inflammation, and decreased hormone excretion^2^.

## Physiological Role of Training

Skeletal muscle has an impressive plasticity which allows it to adapt to the demands placed upon it continuously throughout the lifespan. It is assumed that a higher muscle mass acquired at a young age is a promising starting point to maintain the appropriate muscularity and physical performance in old age. However, hyper-muscularity does not necessarily lead to increased exercise capacity. For example, the myostatin deficient mouse with a hyper-muscular phenotype shows a decreased *in vivo* physical activity^3^. Even so, there is a general agreement that continued moderate physical activity keeps the muscles in good shape. Training of muscle helps to satisfy the complex requirements of elevated activity by increased capillary density and enzyme activity for greater muscle metabolic pretension, as well as elevated production of contractile proteins to produce higher contractile force. On the other hand, sarcopenia is a very complicated mechanism that involves many more phenomena than just the reduction in physical activity with age. The synergy of nutrition and changes in hormone production during the lifetime strongly contribute to the loss of muscle mass and is frequently overlooked. The nature and extent of the contribution of these two factors in keeping the muscle healthy and functional are not yet known.

## Physiological Role of Selenium Treatment

A potential cause of muscle weakness or wasting during aging could be an enhanced nuclear and mitochondrial DNA damage^4^ leading to a decline in muscle fiber numbers and, thus, to impaired skeletal muscle functions. The trace element selenium has an antioxidant property and plays an important role in several muscle functions^5^. In cows, selenium deficiency causes white muscle disease (weakness and degeneration of skeletal and cardiac muscle^6^). In humans, a similar myopathy was also characterized^7^, and insufficient skeletal muscle functions associated with severe selenium deficiency was found^8^. In our previous work on young mice we have shown that selenium supplementation increases calcium release from the sarcoplasmic reticulum (SR) and so improves *in vivo* and *in vitro* skeletal muscle performance. These effects were accompanied by the elevated level of Selenoprotein N in the muscles, which could result in enhanced oxidative stress tolerance during long lasting contractions^9^. As the Selenoprotein N content of the muscle was shown decrease with age^10^, it was of interest to see if long lasting selenium supplementation would reverse this decline and, consequently, improve muscle performance in aged animals.

## Excitation-Contraction (EC) Coupling in Aging Skeletal Muscle

The contraction of muscle is a sequence of well-coordinated processes. The trigger action potential sensed by L-type calcium channels (dihydropyridine receptor, DHPR^11^) in the membrane of the transverse- (T-)tubule, which activate the calcium release channels (type 1 ryanodine receptor, RyR1^12^) in the junctional membrane of the sarcoplasmic reticulum (SR). The released Ca^2+^ initiates muscle contraction, while during relaxation calcium is moved back by the SR calcium pump (SERCA) into the store. The development of this special structural arrangement and the direct coupling is regulated by highly specialized transcription and growth factors and aging could have an impact on these. In aged skeletal muscle fibers the uncoupling between DHPR and RyR1 leads to reduced calcium release from the SR^13^. With aging the area of the SR junctional-face membrane is decreasing^14^ which modifies the level of specific DHPR subunits responsible for the protein-protein interactions involved in EC coupling^15^.

Here we describe the changes of physical performance and excitation contraction (EC)-coupling in aging mice, and show that their alterations in aged animals can be partially or fully reversed not only by training but also by a special diet using selenium as a trace element. On the other hand, increased muscle mass does not, *per se*, have beneficial effects in old age.

## Results

### *In vivo* experiments

To check the *in vivo* muscle performance of the animals, grip tests were used. Control animals showed a decrease in normalized grip force with age, since they gained weight but produced the same maximal force (Table 1). Aged myostatin deficient mice carrying the Compact mutation (*Cmpt*) produced significantly higher maximal force than aged-matched control animals, but after normalization to body weight their performance was significantly lower than in control mice (Table 1). Selenium treatment did not change maximal grip force in old age, but the normalized grip force turned to be significantly higher than in control mice (Table 1). One group of control animals (8 mice) participated in voluntary wheel experiments and were followed from the age of 9 to 90 weeks (corresponding to 2 to 22 months; trained group). As expected, lifelong training increased significantly the maximal and grip force as compared to sedentary aged control animals (Table 1). Furthermore, the body weight was significantly lower thus the normalized grip force was significantly higher in the trained as compared to the control group (Table 1), showing the beneficial effects of physical exercise. Selenium treatment was capable of partially compensating for the lack of training, because the body weight decreased somewhat, but the weight was not significantly different as compared to those of the age-matched control animals (Table 1).

**Table 1:** Body weights and grip force.

	Young	Aged			
	Control (8)	Control (6)	*Cmpt* (7)	Selenium fed (6)	Trained (8)
Body weight (g)	19.7 ± 1.1***	31.2 ± 1.2	52.8 ± 1.8***	28.6 ± 1.0	26.9 ± 0.8**
Maximal grip force (mN)	98.0 ± 2.0	99.5 ± 3.2	138.4 ± 5.1***	104.0 ± 2.9	112.1 ± 3.1**
Maximal grip force normalized to body weight (mN/g)	5.02 ± 0.13***	3.22 ± 0.12	2.61 ± 0.09***	3.63 ± 0.10**	4.17 ± 0.12***

Numbers in parenthesis below the names of animal groups denote the number of animals investigated.

**, and *** denote significant difference compared to aged control at p < 0.01, and p < 0.001, respectively.

**Figure 1:**
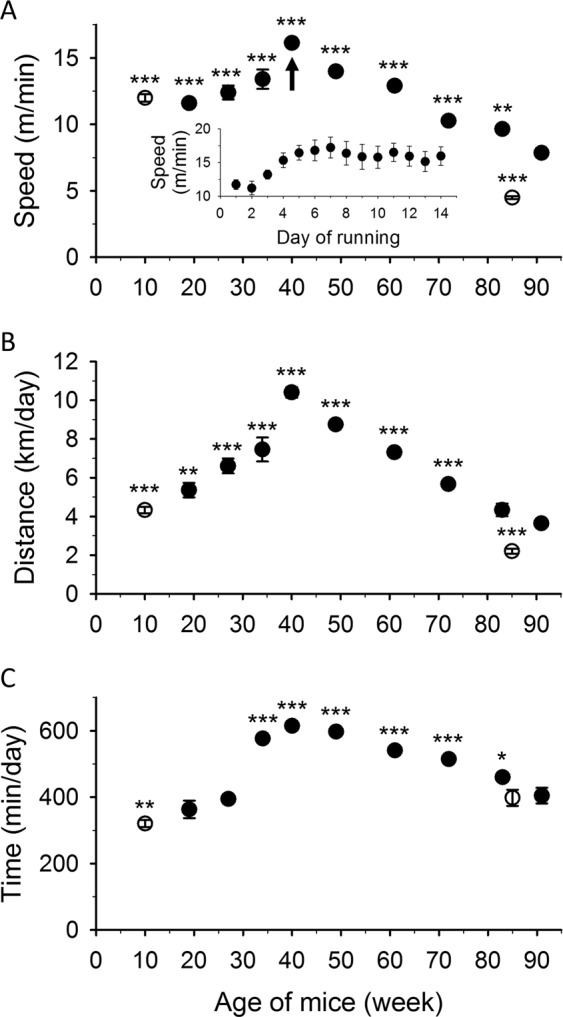
Average speed, daily voluntary running distance and time. Average speed (**A**) daily distance (**B**) and time (**C**) of eight control mice during voluntary running for two weeks in every two months. Empty symbols represent data from measurements on young and aged mice which did not run previously. Inset in panel A shows a typical accommodation of a mouse to the new environment in the cage with the running wheel at the age marked with the arrow. *, **, and *** denote significant difference from the values at 90 weeks of trained aged animals at p < 0.05, p < 0.01, and p < 0.001, respectively.

The voluntary wheel experiments showed age dependent changes in the performance of the animals. Each control animal was placed in a cage with a running wheel and monitored for two weeks every two months from the age of 9 to 90 weeks. After 2–3 days of accommodation in the special cage the average running speed stabilized at a given level (see inset in Fig. 1A). All the other parameters measured (maximal speed, daily running time and distance) followed a similar tendency (data not shown) during the two weeks of running. All parameters of voluntary running showed a decrease from 40 weeks of age in control mice (Fig. 1 and Supplementary Fig. 1). We also performed the voluntary wheel experiment on 21-month-old animals who had not previously run in their lives. The average speed (Fig. 1A), the average maximal speed (Supplementary Fig. 1), and the daily distance (Fig. 1B) was significantly lower in the untrained control group at the age of 85 weeks. However, untrained aged animals spent almost the same time in the running wheel as aged trained mice did (Fig. 1C). Overall, these experiments clearly indicated that there was a negative effect on muscle performance of aging in mice. To clarify the underlying mechanisms, *in vitro* force and the steps in EC-coupling were investigated in detail.

### *In vitro* experiments

#### Force measurements

**Figure 2:**
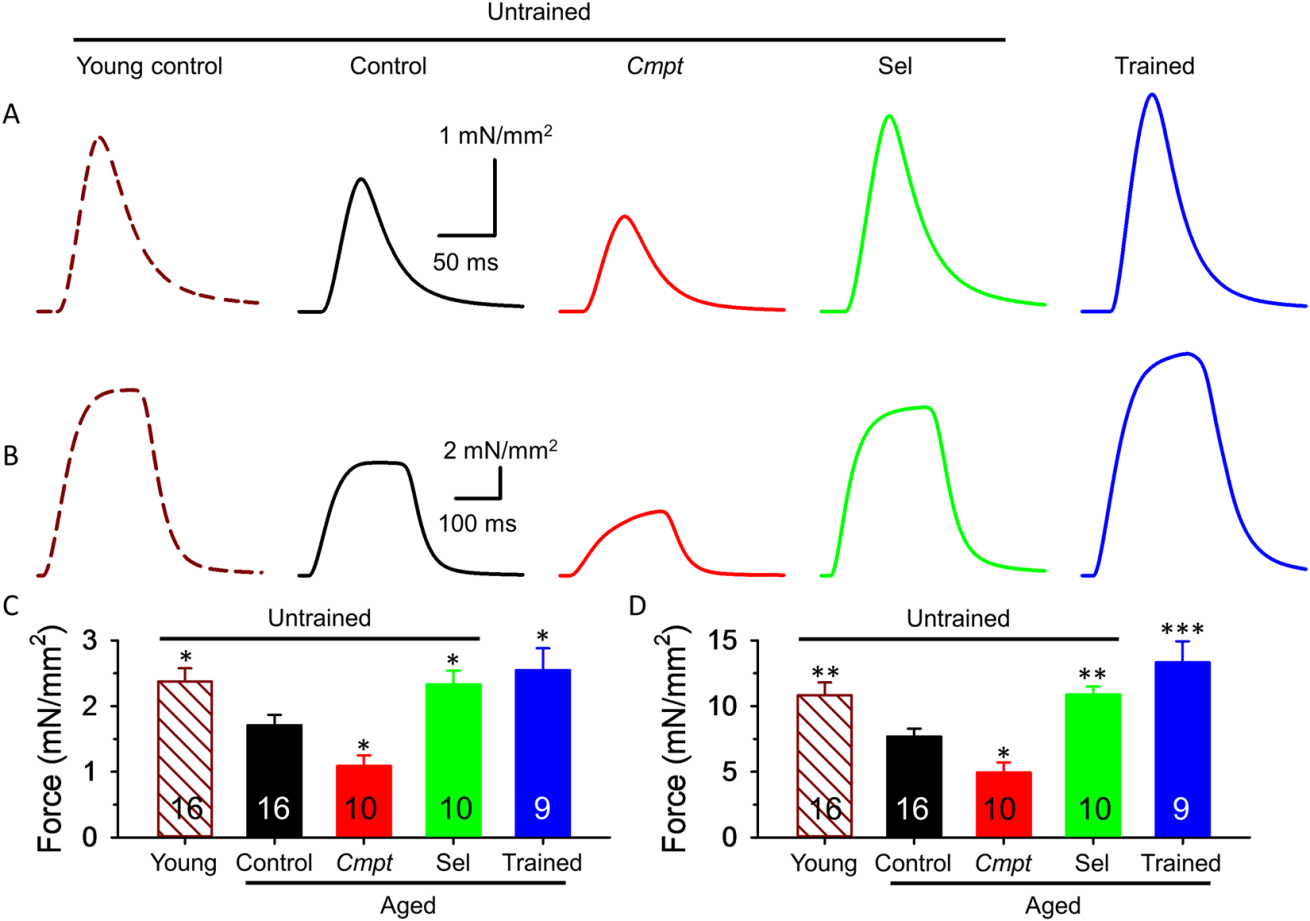
Isometric force in EDL muscle. Twitch (**A**,**C**) and tetanus (**B**,**D**) in EDL muscles of 4-month-old untrained control (brown, dashed line), 20-month-old untrained control (black), *Cmpt* (red), selenium fed (green), and trained control (blue) mouse at room temperature (24 °C). The force was normalized to cross section of the muscle. Numbers in bars give the number of muscles investigated. *, **, and *** denote significant difference from control at p < 0.05, p < 0.01, and p < 0.001, respectively. The number of animals investigated is given in Table 2.

**Figure 3:**
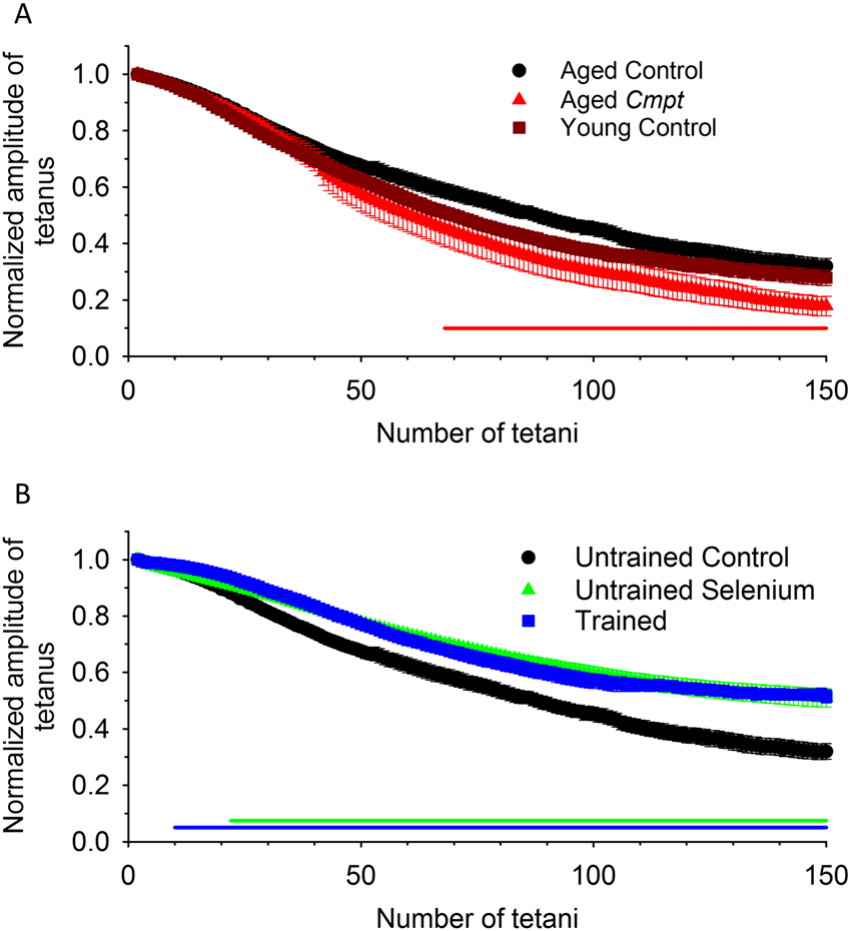
Fatigue measurement. Fatigue of EDL was evoked with 150 tetani at frequency of 0.5 Hz and the amplitude of consecutive tetani was normalized to the first tetanus developed. (**A**) Fatigue was similar in young and aged control mice but significantly higher in aged *Cmpt* than in the other mice. (**B**) Selenium and training increased fatigue resistance in aged mice. Solid horizontal lines below the data points (red: untrained *Cmpt*; green: untrained selenium fed; blue: trained control) represent the interval where data are significantly different from untrained aged control at p < 0.05. The number of muscles investigated is given in Table 2.

As expected, aging decreased *in vitro* muscle force in control animals (Fig. 2). In order to determine whether hypermuscularity, lifelong training, or administration of selenium might alter muscle functions directly, *in vitro* muscle strength was investigated in four groups of aged mice. A significant difference was found in the mean amplitude of the single twitches of *extensor digitorum longus* (EDL) muscle (Fig. 2A,C,) between *Cmpt*, selenium supplemented, trained and the control animals (Table 2). Importantly, selenium supplementation and lifelong training significantly increased, while the *Cmpt* phenotype was associated with a reduced maximal twitch force. A similar significant difference was found in tetanic force as well (Fig. 2B,D). Interestingly, these differences were not present in *musculus soleus* (SOL) (Supplementary Table 1). There were no differences in the twitch/tetanus ratio between the animal groups (Table 2, Supplementary Table 1). Furthermore, selenium and training increased fatigue resistance after 150 tetani in EDL (Fig. 3B, Table 2), but not in SOL (Supplementary Table 1) of aged animals. In contrast, both fast and slow twitch muscles from aged *Cmpt* mice were significantly more fatigable than aged control muscles (Fig. 3A, Table 2, Supplementary Table 1). We did not find significant differences between young and aged control mice in fatigability of EDL (Fig. 3A, Table 2).

**Table 2:** Average parameters of twitch and tetanus of EDL muscles.

Parameters of force		Young	Aged			
		Control (11)	Control (9)	*Cmpt* (7)	Selenium fed (5)	Trained (5)
Peak force (mN/mm^2^)	**Tw**^**§**^	2.38 ± 0.20^*^	1.71 ± 0.16	1.09 ± 0.16^*^	2.33 ± 0.21^*^	2.55 ± 0.33^*^
	**Te**^**§**^	10.83 ± 0.97^**^	7.68 ± 0.61	4.95 ± 0.77^*^	10.89 ± 0.60^**^	13.33 ± 1.61^***^
TTP^**§**^ (ms)	**Tw**	34.6 ± 1.1	32.4 ± 0.7	30.4 ± 1.4	32.7 ± 0.6	32.7 ± 1.0
	**Te**	173.2 ± 4.2	168.8 ± 6.9	164.7 ± 7.0	189.3 ± 3.3^*^	188.2 ± 2.5
HRT^§^ (ms)	**Tw**	29.0 ± 1.0	30.9 ± 1.3	28.0 ± 1.2	29.9 ± 1.4	27.5 ± 1.7^*^
	**Te**	72.8 ± 5.0	75.3 ± 7.4	74.6 ± 5.2	64.2 ± 1.8	62.9 ± 2.5
Duration (ms)	**Tw**	216.5 ± 32.6	185.3 ± 22.8	136.4 ± 6.9	179.2 ± 24.1	132.4 ± 12.2
	**Te**	336.1 ± 4.7	329.7 ± 7.3	322.1 ± 8.6	361.3 ± 9.4^*^	350.2 ± 7.9
Tw/Te		0.20 ± 0.01	0.22 ± 0.03	0.27 ± 0.10	0.21 ± 0.01	0.22 ± 0.01
Fatigue^#^ (%) at tetanus	**50**^**th**^	31.6 ± 2.6	32.5 ± 2.0	40.1 ± 5.5	22.3 ± 1.1^***^	21.5 ± 1.5^**^
	**100**^**th**^	60.4 ± 3.2	54.7 ± 2.4	67.7 ± 4.9^*^	40.5 ± 2.5^***^	43.1 ± 2.6^**^
	**150**^**th**^	67.6 ± 3.7	68.0 ± 2.8	80.0 ± 3.5^*^	49.1 ± 3.3^***^	48.5 ± 2.3^***^
CSA (mm^2^)		1.03 ± 0.06^*^	1.22 ± 0.08	1.90 ± 0.21^***^	0.91 ± 0.05^*^	0.80 ± 0.08^**^
Number of muscles		16	16	10	10	9

Numbers in parenthesis below the names of animal groups denote the number of animals investigated.

*, **, and *** denote significant difference compared to aged untrained control at p < 0.05, p < 0.01, and p < 0.001, respectively. ^#^The amplitude of the 50^th^, 100^th^ and 150^th^ tetanus was normalized to the first tetanus. Fatigue was calculated as 100 · (1 − (normalized tetanus amplitude)). TTP = time to peak; HRT = half relaxation time. ^§^Tw = twitch, Te = tetanus. CSA = cross sectional area of the muscle.

#### Intracellular Ca^2+^ concentration changes (Δ[Ca^2+^]_i_) in aged animals

The effects of muscle hypertrophy, training, and selenium treatment on calcium homeostasis were examined in isolated, single *flexor digitorum brevis* (FDB) muscle fibers from aged mice loaded with the calcium sensitive dye Fura-2 acetoxymethyl (AM). The resting [Ca^2+^]_i_ was significantly higher in *Cmpt* fibers than in fibers from control mice (Table 3). While selenium treatment and training decreased the resting [Ca^2+^]_i_ significantly to the level of young animals (Table 3).

**Table 3:** Average parameters of Ca^2+^ transients and SR Ca^2+^ release in FDB muscle fibers.

	Young	Aged			
	Control (6)	Control (4)	*Cmpt* (6)	Selenium fed (6)	Trained (3)
Resting [Ca^2+^]_i_ (nM)	71.5 ± 0.8***	78.1 ± 1.6	88.3 ± 4.9**	68.1 ± 2.1***	70.0 ± 1.5*
Δ[Ca^2+^]_i_ (μM)	643.7 ± 63.6	566.3 ± 62.4	163.4 ± 24.1***	1080.3 ± 193.5**	1712.7 ± 235.3***
Ca^2+^ release flux (μM/s)	644.7 ± 41.7**	481.2 ± 34.0	186.8 ± 31.3***	800.6 ± 80.7***	1116.5 ± 66.3***
Amount of Ca^2+^ released (μM)	1332.2 ± 92.7	1326.6 ± 112.5	426.7 ± 81.0***	1355.6 ± 202.7	1716.5 ± 159.5**
Number of fibers	39	25	11	14	8

Numbers in parenthesis below the names of animal groups denote the number of animals investigated.

*, **, and *** denote significant difference p < 0.05, 0.01, and 0.001 compared to aged control.

**Figure 4:**
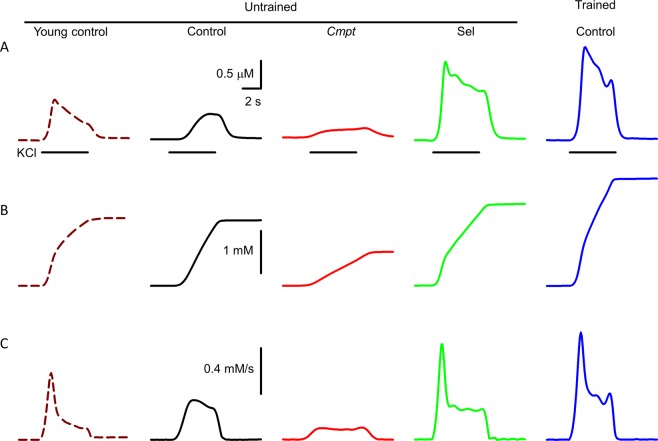
KCl depolarization-evoked Ca^2+^ transients and SR Ca^2+^ release in FDB fibers. Depolarization- (KCl) evoked changes in intracellular calcium concentration (**A**), amount of Ca^2+^ released (**B**), and Ca^2+^ flux (**C**) in FDB muscle fibers of 4-month-old untrained control (brown, dashed line), 20-month-old untrained control (black), *Cmpt* (red), selenium fed (green), and trained control (blue) mouse. Black horizontal lines below the calcium transients represent the application of KCl.

To assess the SR calcium content – the amount of releasable calcium from the SR – 5-second-long depolarizations using 120 mM KCl were applied. Figure 4A shows representative calcium transients evoked by such depolarizations in the presence of normal (1.8 mM) extracellular calcium concentration. Selenium and training increased the amplitude of the calcium transients, while it was smaller in *Cmpt* than in control mice. Pooled data in Table 3 confirm that selenium supplementation and training have statistically significant effects on increasing the change in [Ca^2+^]_i_, suggesting that the calcium release from the SR was higher under these conditions. On the other hand, depolarization-evoked calcium transients were notably smaller in *Cmpt* than in control fibers (Fig. 4A, Table 3). Selenium almost doubled, and training almost tripled the amplitude of the calcium transients. Further examination of the transients proved that the amount of releasable calcium from the SR was significantly higher after training (Fig. 4B, Table 3), and significantly lower in *Cmpt* than in untrained control fibers, while unchanged following selenium treatment. It has to be noted that while the amount of calcium released form the SR was unaltered in selenium fed mice the amplitude of the calcium transient was significantly elevated. This suggested a modified kinetics of SR calcium release which was confirmed by calculating SR Ca^2+^ flux that proved to be significantly greater in both selenium fed and trained, while significantly lower in *Cmpt* than in untrained control mice (Fig. 4C, Table 3).

#### Protein composition of aged muscle

**Figure 5:**
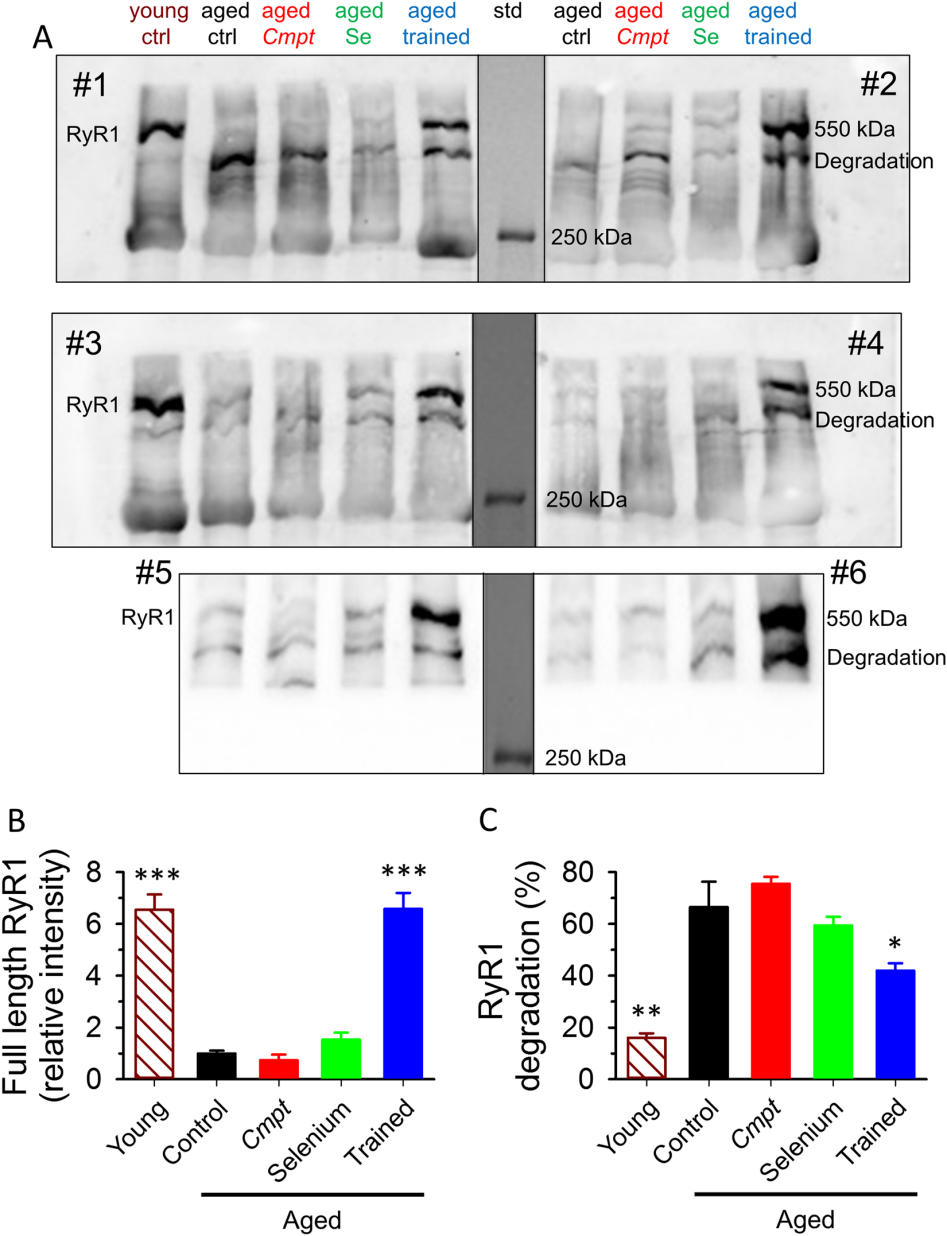
Expression of RyR1 in aged muscle. Representative membrane of immunoblots stained by specific RyR1 antibodies from 8 aged mice (2 in each group) and from one young animal (**A**). The middle, black vertical lines indicate the merging border between standard (std) and bands. The blot was stained with an antibody from Thermo Scientific. Additional raw unmodified membranes are shown in Supplementary Fig. 2. Averaged RyR1 (550 kDa band) content normalized to aged control from 4 young and 6 aged mice from each group (**B**). Averaged percentage of degraded RyR1 presents in all Western blots (**C**). *, and *** denote significant difference from untrained aged control at p < 0.05 and p < 0.001, respectively.

**Figure 6:**
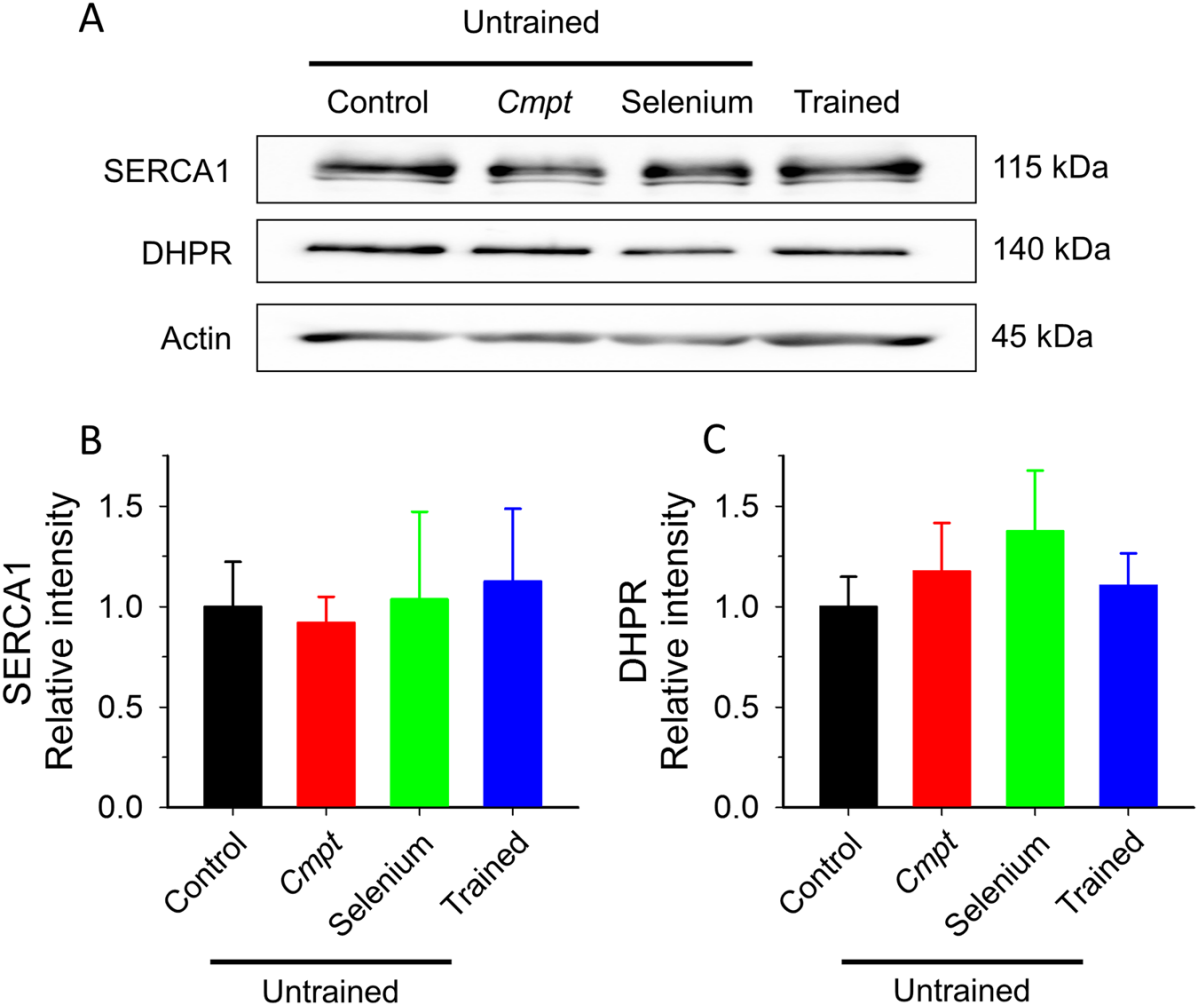
Expression of SERCA1 and DHPR in aged muscle. Representative Western blot images showing the expression level of SERCA1, and DHPR in *quadriceps femoris* muscles (**A**). Actin was used as loading control. Averaged content of SERCA1 (**B**), and DHPR (**C**) in muscles from 4 aged mice in each group normalized first to actin and then to aged untrained control. Raw, unmodified membranes are shown in Supplementary Fig. 4A.

The expression pattern of key proteins of EC-coupling were also investigated. Figure 5A shows representative Western blot from all animal groups (additional blots are presented in Supplementary Fig. 2). Interestingly, RyR1 showed a strong age-dependent decrease in all untrained animal groups, but not in trained control mice (Fig. 5B). In particular, lifelong running conserved full length RyR1 content at the level of young animals (Fig. 5B). On the other hand, significantly increased amount of a smaller molecular weight degradation product of RyR1 could be observed in aged animals (Fig. 5C). Whereas, lifelong training resulted in a significantly less degradation of the normal, 550 kDa protein (Fig. 5C). The expression pattern of RyR1 was confirmed with two different antibodies (Supplementary Table 2). There was no significant difference in the normalized levels of SERCA and DHPR between the muscles from different groups of aged animals (Fig. 6). Furthermore, we did not find significant difference in the amount of DHPR and SERCA between young and aged control animals (Supplementary Fig. 3).

**Figure 7:**
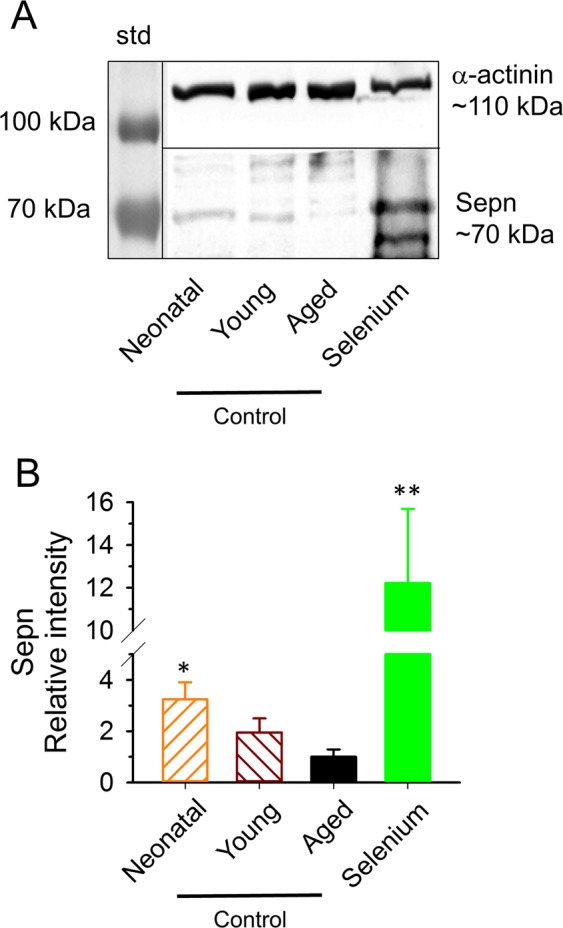
Age dependent Selenoprotein N expression in C57BL/6 mice. (**A**) Representative immunoblots stained by specific Selenoprotein N (Sepn) antibody (see Supplementary Table 2) in *quadriceps femoris* muscle from a 4-day-old (Neonatal), a 4-month-old (Young), a 22-month-old (Aged) control and a selenium supplemented (Selenium) mouse. The left lane is the standard (std). α-actinin was used as loading control. (**B**) Averaged relative intensity from 3 membranes (12 mice, 3 from each group). *, and ** denote significant difference from aged control at p < 0.05 and p < 0.01, respectively. Raw, unmodified membranes are shown on Supplementary Fig. 4D. Black vertical and horizontal lines indicate the merging border between standard (std) and the bands, and the bands detected with different exposition times, respectively.

As the trace element selenium proved to beneficial in preventing age-related decline in muscle performance (as demonstrated above) and was shown to increase Selenoprotein N content in our earlier studies^9^ it was of interest to see how Selenoprotein N (Sepn) expression is changing with age and, if yes, whether selenium supplementation could counteract this change. The 70 kDa Sepn content showed a gradual decrease with age, declining to 31% in 22-month-old mice as compared to that of that measured in neonatal animals (Fig. 7). Following the selenium supplemented diet a remarkable elevation in the level of Selenoprotein N was observed (Fig. 7). In parallel, the average selenium content of EDL muscles of aged mice was determined and was found to be significantly higher in the muscles of selenium fed (197.1 ± 6.6 ng/g, n = 4, p < 0.05) animals than in controls (140.5 ± 1.8 ng/g, n = 4).

#### Increased ROS production in aged muscle

**Figure 8:**
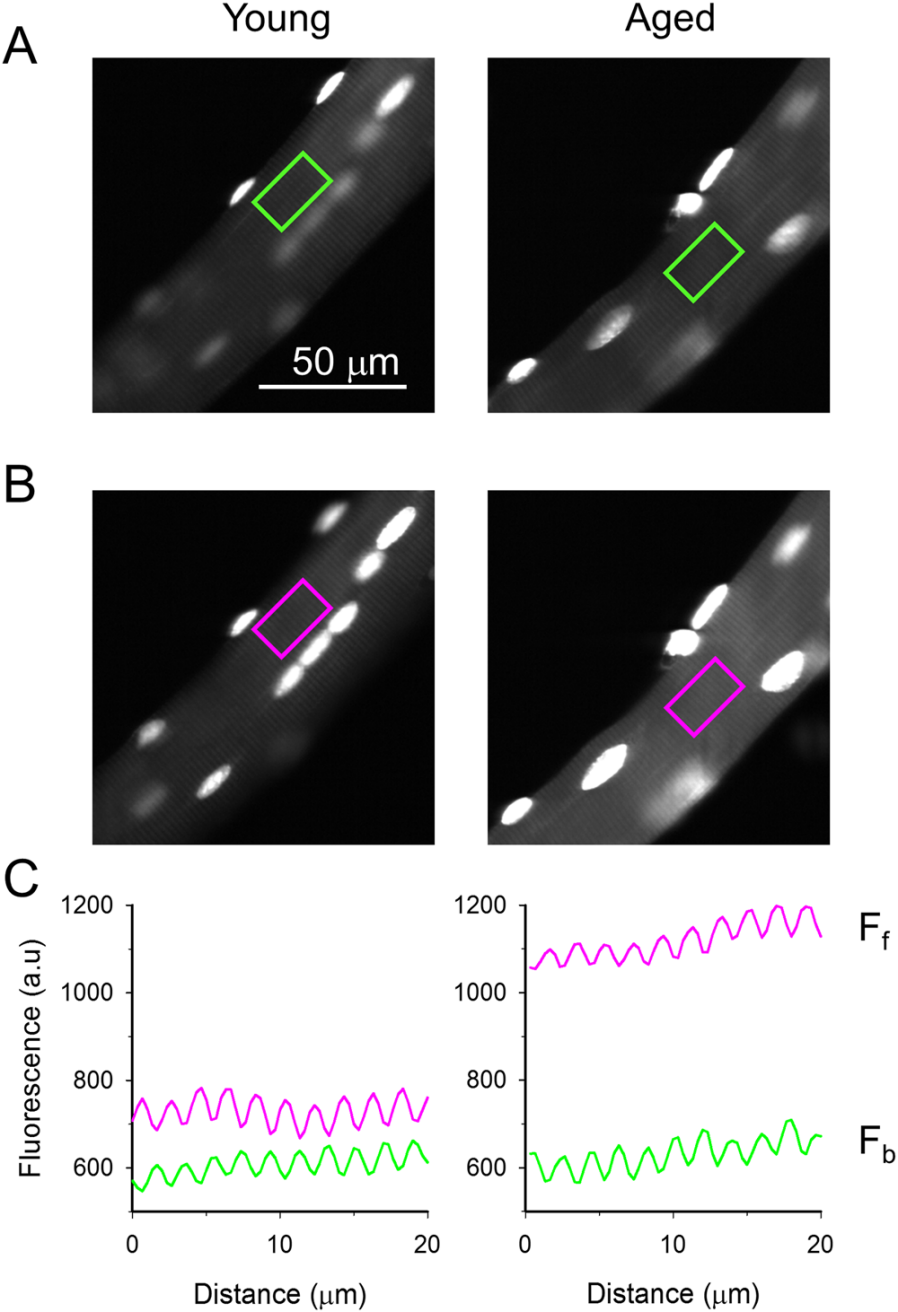
ROS measurement. Fluorescence intensity of a resting FDB fiber loaded with 3 nM Dihydroethidium excited at 532 nm and detected at >550 nm (**A**). The same fiber after field stimulation with a series (150) of tetani evoked by 2 ms long square pulses with 50 Hz frequency (**B**). Background corrected fluorescence values averaged over the area marked with a rectangle on panels A (green; fluorescence before stimulation - F_b_) and B (pink; fluorescence following stimulation - F_f_) in parallel with the longitudinal axis of the fiber (**C**). Note that the periodic increase and decrease in fluorescence represent the sarcomeric pattern of dye distribution.

As RyR1 was suggested to be influenced by oxidative stress and selenoproteins are assumed to serve as guardians against ROS^5^, our above results suggested that muscles of aged animals might be exposed to increased oxidative stress than those from young mice. To confirm this hypothesis the oxidative status of the muscle fibers was examined by measuring ROS production. To this end isolated skeletal muscle fibers from FDB muscles of young (4-month-old) and aged (22-month-old) mice were loaded with Dihydroethidium and the dye fluorescence was recorded before (F_b_) and following (F_f_) a fatiguing protocol that consisted of a series of 150 tetani evoked by 2 ms long square pulses with 50 Hz frequency (Fig. 8). ROS production was quantified by calculating the ratios of background corrected fluorescence (F_f_/F_b_) and was found to be significantly higher in aged (1.54 ± 0.14, n = 4, p < 0.05) than in young (1.11 ± 0.07, n = 6) muscle fibers after the fatiguing protocol, confirming the notion that aged muscles are exposed to an elevated oxidative stress.

## Discussion

The results presented here provide novel data regarding the effects of dietary selenium and lifelong training on skeletal muscle performance in aging.

### Effects of aging on muscle performance

A considerable loss of muscle strength and mass (sarcopenia) with increased fatigability, impaired regenerative capacity, and moderate physical achievement are associated with aging skeletal muscle^16–18^. While the pathophysiology of sarcopenia is characterized by increased oxidative stress and decreased hormone production, recent evidence suggests that elevated myostatin levels may also contribute to this process^19^. Since we did not observe any positive effects on muscle performance in aged *Cmpt* mice, a mouse strain with reduced myostatin expression, our results suggest that changes in the myostatin levels do not have a significant influence on muscle performance in aged individuals. Remarkably, physical activity and exercise might prevent or compensate for muscle aging, and have been reported to weaken age-related decrease in muscle strength, mass, and regenerating capability and, moreover, to prevent pathological alterations in muscle metabolism^18^. The impaired force per cross-sectional area (specific tension) is a major contributor to the decreased intrinsic muscle tension. Subsequent studies have shown that this is rather a cause of a decreased number of actomyosin cross-bridges, than the reduced force developed by a single cross-bridge^20^.

It should also be acknowledged that decreased physical performance with aging is partly due to the decline in cardiovascular capacity (for review see^21^). The aging heart shows increased ventricular hypertrophy and decline in exercise tolerance due to impaired maximal heart rate. These are accompanying with reduced oxygen extraction because of the decreased peripheral muscle mass.

### Effects of long term training on muscle performance

The positive effects of routine modest exercise have been reported in several studies on humans and with experimental animals (for review see^22^). It has been shown that chronic exercise slightly increases the lifespan of rats^23^, and similar results of moderate exercise were observed in mice^24^. To assess the beneficial effects of regular modest exercise at a whole-body level, the changes in the neurological responses with aging was determined in mice^22^. Long term exercise improved mouse performance in physical tests, showing a higher quality of neuromuscular and neurological processes. Several clinical trials proved the positive effects of exercise by lowering hypertension^25^, hypercholesterolemia^26^, and hyperglycemia^27^. However, how physical exercise leads to these positive effects is still poorly understood. One possible candidate is muscle adaptation to routine exercise by enhancing mitochondrial functions and synthesis of new elements of the respiratory chain to feed the increased energy necessity^28^. Studies have indicated that ROS production can activate signaling pathways that mediate skeletal muscle adaptations to exercise^29,30^. One can, therefore, hypothesize that lifelong training could serve as a pre-conditioning for ROS tolerance in aged muscle. We have indeed shown here and others^24^ previously the beneficial effects of routine training on physical performance in aged mice. Two possible pathways for these effects - both associated with oxidative stress - should be considered. i) Since RyR1 is very sensitive to oxidative changes, its accommodation to oxidative stress through conditioning with lifelong training could improve the calcium channel functions as demonstrated by the decreased degradation of RyR1 in trained animals. ii) As RyR1 degradation was not reduced in selenium supplemented aged animals, nevertheless selenium as an antioxidant increased muscle performance both in young^9^ and aged animals (this study), RyR1 independent pathway(s) should also underlie the observed effects.

### RyR1 expression in aged muscle

An impaired EC-coupling may also contribute to the reduction in muscle strength, as there is increasing evidence of EC-uncoupling with aging, since a reduction in DHPR’s function leading to decreased Ca^2+^ release from the SR has been reported in aging skeletal muscle^31,32^. Furthermore, phosphorylation and redox-dependent modifications of RyR1 have also been associated with abnormal Ca^2+^ homeostasis and contractile dysfunction in long lasting muscle fatigue^33^. Defective SR Ca^2+^ release has been reported in age-dependent muscle weakness^34^. Here, we demonstrate a decreased RyR1 content in aged muscle which could be compensated by lifelong training. Interestingly, in parallel with the decreased RyR1 we observed an increased amount of the degraded calcium channel protein (see Fig. 5) in all aged animal groups. This degradation of RyR1 was also observed earlier by another group^35^. While this was not completely reversed in the trained aged animals, the intact RyR1 was present at a level comparable to that found in young mice (Fig. 5). One can thus speculate that the continuous usage of the muscles prevents the otherwise inevitably increased degradation of RyR1 in old age, which consequently helps to maintain the necessary number of operable calcium release channels.

### Mismatch between muscle mass and contractile force

Our results clearly demonstrate that *Cmpt* mice have greater muscle mass than control animals do and this persists into old age. This observation goes hand in hand with a greater maximal *in vivo* force. However, when the *in vivo* force is normalized to body weight it is significantly lower in the *Cmpt* animals (Table 1), despite the fact that the difference in body weight results from an increase in muscle mass. Similar results were obtained in myostatin knockout mice previously^36^. Moreover, the physical performance and, consequently, *in vitro* fatigue are more pronounced in *Cmpt* mice (Fig. 3). All these findings point in the direction that muscle performance not only decays in parallel with aging as expected from a number of previous observations but greater muscle mass at a younger age doesn’t necessarily result in better performance in aged mice. This is in line with several publications that show that muscle mass and strength do not necessarily correlate with each other during aging and muscle fatigue is also affected in old age (for review see^37^). On the other hand, human studies demonstrated that muscle performance not only declines progressively with aging but can also be affected by neurological factors and obesity^38^. It should of course also be noted that in *Cmpt* mice the cardiovascular system is unable to suffice with the increased metabolic demand and maintain the normal circulation against the higher peripheral resistance of the vasculature. Furthermore myostatin has been shown to be a pro-oxidant and increase ROS production in skeletal muscle^39^, thus *Cmpt* mice could have an increased basal antioxidant enzyme level and more efficient scavenging of excess ROS. All these point in the direction that the interpretation of myostatin inhibition and consequently the results from *Cmpt* mice might not be straightforward.

### The effects of selenium on muscle performance

Selenium is expansively used as a dietary complement, either alone or along with vitamins. We have shown previously that it has beneficial effects on muscle performance in young mice^9^. A two week selenium treatment improved maximal voluntary running speed, and fatigue resistance. In the present study we found similar beneficial effects on force in the EDL and SOL of aged mice following an 8-week-long application of selenium. Fatigue resistance was significantly improved in EDL (Fig. 3), and albeit a similar tendency was observed in soleus (Supplementary Table 1) it did not proved to be significant. As aging specifically affects IIB type fibers^40^ and EDL contains more IIB fibers than SOL it is expected that selenium supplementation or lifelong training would affect the former more than the latter. Since these beneficial effects on muscle performance were not accompanied by atrophy or any change in the expression level of the key proteins involved in EC-coupling, selenium might be involved in the metabolic chain during contraction. This is substantiated by a mediation analysis (see Supplementary Fig. 5) which suggests that the effects of selenium supplementation are not mediated through changes in intracellular calcium concentration. It is known that reactive oxygen and reactive nitrogen species play a crucial role in the plasticity of skeletal muscle during exercise and disuse. They are produced in the sarcolemma, transverse tubules, SR, cytosol, and in mitochondria^41^, but the source of exercise-induced superoxide is still debated. It has recently been shown that selenium has a positive antioxidant effect in chicken skeletal muscle during heat shock^42^. During long lasting contractions oxidants could accumulate in the muscle, leading to decreased force production. Indeed, a tetanic stimulation protocol increased ROS production more in muscles from aged as compared to young animals (Fig. 8). This suggests that the reduced force in aged untrained animals seen in our experiments could be, at least partially, due to an increased oxidative stress. Interestingly, increased fatigue tolerance associated with selenium supplementation was comparable to that of lifelong training. This effect of selenium can be a very useful in old age when muscle mass is decreased. In addition, unlike lifelong training, selenium supplementation did not alter SR Ca content but increased the maximal rate of Ca efflux from the SR. This suggests that increased levels of Sepn prevented, at least partially, the oxidative modification of RyR.

### Selenium supplementation and selenoprotein content in old age

Previous trials tested the effects of selenium in old age^43^, and their results suggest that antioxidant supplementation is capable of improving the capability of leucine to activate the muscles to synthetize proteins in aged rats. This process can be mediated by reducing oxidative stress. Similar results were obtained when mice were treated with increased levels of a special antioxidant mixture containing selenium^44^. The overall oxidative state of mice was improved, accompanied with a decreased muscle fatigue and increased maximal grip force. As the consequence of antioxidant supplementation, an enhanced mitochondrial function as the consequence of more potent mitochondrial dynamics was hypothesized by the authors. We also found beneficial effects of selenium on both *in vivo* and *in vitro* muscle function and, in addition, on calcium homeostasis in aged mice. The fact that selenium has similar positive effects on muscles from young^9^ and aged animals (this study) underlines its potential application in the treatment of sarcopenia.

Several studies including ours proved that selenium supplementation can increase selenoprotein expression^9^. It was also shown that selenoprotein expression decreases with age^10^. We also found a decreased Selenoprotein N content in skeletal muscles of aged mice which was reversed by the 2-month-long selenium supplementation (Fig. 7).

Taken together, our results, on the one hand, provide evidence that – unlike moderate training – increased muscle mass does not, *per se*, prevent age-dependent decline in muscle force and in normal RyR1 content. On the other hand, they suggest that an antioxidant strategy in the form of selenium supplementation in the diet could be a suitable therapeutic procedure for patients not only with myopathies, but also with aging.

## Materials and Methods

### Animal care and experimental design

Animal experiments conformed to the guidelines of the European Community (86/609/EEC). The experimental protocol was approved by the institutional Animal Care Committee of the University of Debrecen (31/2012/DE MAB). Mixed sex mice were housed in plastic cages with mesh covers, and fed with pelleted mouse chow and water *ad libitum*. Room illumination was an automated cycle of 12 h light and 12 h dark, and room temperature was maintained within the range 22–25 °C.

C57BL/6 mice received normal diet and they performed voluntary activity for 14 days in every 2 months in a running wheel cage as reported earlier^9^ from the age of 2 months until the end of their life (hereafter referred to as *control trained*). In the second (4-month-old,) and third (20–22-month-old) groups C57BL/6 mice received normal diet and were kept in normal mouse cages, *i.e*. they did not perform regular voluntary activity (referred to as *untrained young control* and *untrained aged control*, respectively). In the fourth group 20–22-month-old *Cmpt* mice^45^ received normal diet, were kept in normal mouse cages, and did not perform regular voluntary activity (referred to as *untrained Cmpt*). In the fifth group 20–22-month-old C57BL/6 mice followed the same protocol as those in the untrained aged control group but received a selenium supplemented (see below) diet (referred to as *untrained Selenium fed*).

The selenium feed used here was described previously^9^. In brief, elementary nano-sized selenium (NanoSe) was administered for 2 months to 20–22-month-old untrained control mice at concentrations of 5 particle per million Se (equivalent to 5 mg Se/kg food, corresponding to an estimated 40 µg/kg body weight/day Se uptake) before using them for experiments. Production of nano-sized (100 to 500 nm diameter) elemental selenium (NanoSe) was described earlier^46^. The estimation of the total selenium content of the EDL muscle was described earlier^47^.

### *In vivo* experiments

#### Voluntary activity wheel measurement

Mice from the different groups (see above) were singly housed in a cage with a mouse running wheel (Campden Instruments Ltd., Loughborough, UK). Wheels were interfaced to a computer and revolutions were recorded in 20 minutes intervals, continuously for 14 days. The daily average and the maximal speed, the distance and the duration of running were calculated for each individual mouse and then averaged by groups.

#### Forepaw grip test

The force of forepaw was measured as described earlier^3^. Briefly, when the animals reliably grasped the bar of the grip test meter, they were then gently pulled away from the device. The maximal force before the animal released the bar was digitized at 2 kHz and stored by an online connected computer. The test was repeated 10–15 times to obtain a single data point. Measurements for the trained groups were always carried out before the 14 days running regime. For all other animal groups grip test was measured on the day when the animals were sacrificed.

### *In vitro* experiments

Animals were anaesthetized and sacrificed following a protocol approved by the Animal Care Committee of the University of Debrecen (31/2012/DE MAB). After pentobarbital anesthesia (27 mg/kg) and cervical dislocation, the *m. flexor digitorum brevis* (FDB), the *m. extensor digitorum longus* (EDL), *m. soleus* (SOL) and *m. quadriceps femoris* muscle from the hind limb were dissected.

#### Measurement of muscle force

Muscle contractions were measured as described in our previous report^9^. In brief, fast and slow twitch muscles, EDL and SOL, were removed and placed horizontally in an experimental chamber continuously superfused (10 ml/min) with Krebs’ solution (containing in mM: NaCl 135, KCl 5, CaCl_2_ 2.5, MgSO_4_ 1, Hepes 10, glucose 10, NaHCO_3_ 10; pH 7.2; room temperature) equilibrated with 95% O_2_ plus 5% CO_2_. One end of the muscle was attached to a capacitive mechano-electric force transducer (Experimetria, Budapest, Hungary). Two platinum electrodes placed adjacent to the muscle were used to deliver short, supramaximal pulses of 2 ms in duration at 0.5 Hz to elicit single twitches. To elicit a tetanus, single pulses were applied with a frequency of 200 Hz for 200 ms (EDL) or 100 Hz for 500 ms (SOL). Fatigue was evoked with 150 tetani at frequency of 0.5 Hz and the amplitude of consecutive tetani was normalized to the first tetanus developed. Muscles that were visibly injured during the manual preparation were excluded from the evaluation.

#### Isolation of single skeletal muscle fibers

All calcium concentration measurements were carried out on skeletal muscle fibers from the FDB muscle of the mouse. Calcium free Tyrode’s solution (containing in mM, 137 NaCl, 5.4 KCl, 0.5 MgCl_2_, 5 ethylene glycol-bis (β-aminoethyl ether)-N,N,N’,N’-tetraacetic acid (EGTA), 11.8 HEPES; 1 gL^−1^ glucose; pH 7.4) was used during the dissection of the muscle. Single muscle fibers from FDB were enzymatically dissociated in minimal essential media containing 0.2% Type I collagenase (Sigma) at 37 °C for 65 minutes^48,49^. To release single fibers, the FDB muscles were triturated gently in normal Tyrode’s solution (1.8 mM CaCl_2_, 0 mM EGTA). The isolated fibers were then placed in culture dishes and stored at 4 °C in refrigerator until use.

#### Whole cell intracellular Ca^2^+ concentration measurement

Changes in intracellular Ca^2+^ concentration ([Ca^2+^]_i_) were monitored using Fura-2 AM as described previously^3,49^. Briefly, isolated FDB fibers were mounted on a laminin-coated cover slip and loaded with 5 µM Fura-2 AM for 60 min. Fibers were then equilibrated in Tyrode’s solution for 30 minutes at room temperature. The excitation wavelength was altered between 340 and 380 nm by a microcomputer-controlled dual-wavelength monochromator (Deltascan, Photon Technology International, New Brunswick, NJ), whereas the emission was monitored at 510 nm using a photomultiplier at 10 Hz acquisition rate of the ratios at 22 °C. Ca^2+^ transients were evoked by KCl depolarization. Intracellular calcium concentration was calculated from the ratio of measured fluorescence intensities using an *in vivo* calibration as described in our earlier report^49^.

To determine the Ca^2+^ released into the myoplasm, the Ca^2+^ binding to intracellular binding sites and the Ca^2+^removal from the intracellular space (the activity of the Ca^2+^ pump) was modeled as presented in earlier report^50^. In brief, Ca^2+^ binding to intracellular sites – to the SERCA pump (P), to troponin C (TnC), to parvalbumin (parv) and to the dye – and the removal were considered. The maximal transport rate of the Ca^2+^ pump from the declining phase of the Ca^2+^ transient following the stimulation was determined as a single best fit parameter. All other parameters in the model were held constant at values [parv] = 690 µM, K_on,Ca-parv_ = 160 µM^−1^ s^−1^, K_off,Ca-parv_ = 1.5 s^−1^, K_on,Mg-parv_ = 0.4 µM^−1^ s^−1^, K_off,Ca-parv_ = 8 s^−1^, [trop] = 250 µM, K_on,Ca-TnC_ = 20 µM^−1^ s^−1^, K_off,Ca-TnC_ = 150 s^−1^, [P] = 200 µM, P_KD_ = 0.5 µM. The Ca^2+^ flux was calculated as the first time derivative of the amount of Ca^2+^ released. Note that the amount released with such long depolarization (5 s) is a good estimate of the SR calcium content^51^.

#### Western blot analysis

*Musculus quadriceps femoris* tissue samples from the hind limb were homogenized in lysis buffer (20 mM Tris–Cl, 5 mM EGTA, protease inhibitor cocktail (1:100) all from Sigma) and disrupted by sonication on ice. The protein content of the samples was measured by a modified bicinchoninic acid protein assay (Pierce, Rockford, IL, USA) using bovine serum albumin as a standard. For Western-blot analysis (SDS–PAGE) total muscle lysates were prepared by adding 1/5 volume of five-fold concentrated electrophoresis sample buffer (310 mM Trise-HCl pH 6.8, 10% SDS, 50% glycerol, 100 mM dithiothreitol (DTT), 0.01% bromophenol blue) to samples and boiled for 10 min at 80 °C. 10 μg of protein was separated by 5% and 7.5% SDS–PAGE gel for immunological detection of examined proteins. Proteins were transferred electrophoretically to nitrocellulose membranes. After blocking with 5% non-fat dry milk in phosphate-buffered saline (PBS), membranes were incubated with the appropriate primary antibodies overnight at 4 °C (see Supplementary Table 2). After washing three times for 10 min with PBST (PBS supplemented with 0.1% Tween 20), membranes were incubated with a secondary antibody, peroxidase-conjugated goat anti-rabbit, and anti-mouse immunoglobulin G (Bio-Rad) in 1:1000 dilution in PBS containing 5% non-fat dry milk for 1 h. Signals were detected by enhanced chemiluminescence (ECL) reaction (Thermo Scientific, Rockford, IL, USA). The intensity of the specific bands and the background from the same image were measured by ImageJ (NIH, Bethesda, MD, USA). The background corrected normalized values were given as relative to the intensity of the housekeeping genes in the same samples. These data were then expressed as relative to control.

#### ROS measurements

Single FDB fibers were loaded with 3 nM Dihydroethidium for 20 minutes at room temperature. Fluorescence intensity of resting fibers was measured by exciting at 532 nm and detecting at >550 nm. The measurement was repeated on the same fiber after field stimulation with a series (100) of tetani evoked by 2 ms long square pulses with 50 Hz frequency for 100 ms. The background corrected fluorescence values were averaged over a selected area parallel with the longitudinal axis of the fibers. The calculated curves showed a periodic increase and decrease in fluorescence representing the sarcomeric pattern of dye distribution. The average values of the curves were assumed to be proportional with the ROS production in the fibers.

#### Statistical analysis

Pooled data were expressed as mean ± standard error of the mean (SEM). The mean and SEM were calculated as weighted averages and weighted standard errors with weights corresponding to the number of muscles from the same animal, while the number of samples was the number of animals in the given group. The differences between animal groups were assessed using one way analysis of variance (ANOVA) and all pair wise multiple comparison procedures (Bonferroni’s multiple comparisons test) using the statistical program Prism (GraphPad Software, San Diego, CA, USA). A p value of less than 0.05 was considered statistically significant. A causal mediation model was constructed to examine whether the association of selenoprotein with maximal tetanic force was mediated by intracellular Ca^2+^ concentration. Details of statistical tests are presented in Supplementary Information.

## Supplementary information

Supplementary information.

## Data Availability

The datasets generated and analyzed during the current study are available from the corresponding author on reasonable request.
