# Supplementary material for: Improved Calcium Homeostasis and Force by Selenium Treatment and Training in Aged Mouse Skeletal Muscle
Source: Sci Rep. 2020 Feb 3;10:1707. doi: 10.1038/s41598-020-58500-x (PMC6997352; doi:10.1038/s41598-020-58500-x)
Supplement: Supplementary file 1 — Supplementary information. [file 41598_2020_58500_MOESM1_ESM.pdf]

IMPROVED CALCIUM HOMEOSTASIS AND FORCE BY SELENIUM TREATMENT AND TRAINING IN AGED MOUSE SKELETAL MUSCLE

SUPPLEMENTARY INFORMATION

[1,3]János Fodor, [1,2,3]Dána Al-Gaadi, [1,2]Tamás Czirják, [1]Tamás Oláh, [1]Beatrix Dienes, [1]László Csernoch and [1]Péter Szentesi[*]

[1]Department of Physiology, Faculty of Medicine, University of Debrecen, Debrecen, Hungary

[2]Doctoral School of Molecular Medicine, University of Debrecen, Debrecen, Hungary

[3]These authors contributed equally to this article.

Running title: Effects of training and selenium on aging skeletal muscle functions

Key words: skeletal muscle; intracellular calcium; force; aging; training; selenium;

[*] - Author for correspondence: Dr. Péter Szentesi, Department of Physiology, Faculty of Medicine, University of Debrecen, P.O. Box 400, H-4002 Debrecen, Hungary; Tel: +36-52-255575, Fax: +36-52-255116, E-mail: szentesi.peter@med.unideb.hu

János Fodor	fodor.janos@med.unideb.hu
Dána Al-Gaadi	al-gaadi.dana@med.unideb.hu
Tamás Czirják	czirjak.tamas@med.unideb.hu
Tamás Oláh	olahtamas@gmail.com
Beatrix Dienes	dienes.beatrix@med.unideb.hu
László Csernoch	csl@edu.unideb.hu

Supplementary Table 1. Average parameters of twitch and tetanus in soleus muscles

Parameters of force		Young control n=11	Aged untrained control n=9	Aged untrained *Cmpt* n=7	Aged untrained selenium fed n=5	Aged trained control n=5
**Peak force (mN/mm^2)**	**Tw**[§]	2.06±0.26	2.03±0.36	1.83±0.41	2.07±0.29	2.01±0.22
	**Te**[§]	11.36±1.46	10.21±1.62	11.07±1.33	10.06±1.31	10.92±0.88
**TTP[§] (ms)**	**Tw**	70.5±4.3	64.7±3.5	68.2±4.2	81.1±2.5**	97.7±6.2***
	**Te**	505.9±2.8**	516.0±2.2	520.0±7.8	521.3±1.7	526.6±2.6**
**HRT[§] (ms)**	**Tw**	67.1±5.8	63.3±3.5	65.4±5.8	73.4±3.9	86.1±4.4*
	**Te**	91.5±2.3***	126.2±6.7	109.5±5.6	149.7±3.1*	119.4±6.0
**Duration (ms)**	**Tw**	311.1±22.2	286.9±18.2	273.1±18.8	306.7±15.9	364.8±25.3*
	**Te**	701.6±7.9***	820.9±25.5	796.6±34.4	862.6±13.9	780.8±13.6
**Tw/Te**		0.20±0.03	0.22±0.04	0.15±0.02	0.21±0.02	0.20±0.04
**Fatigue[#] (%) at tetanus**	**50th**	33.5±4.2*	22.8±0.7	34.9±3.9**	20.9±2.3	29.7±1.0***
	**100th**	54.6±4.5*	40.7±1.8	60.1±4.0***	36.6±3.9	44.6±2.2
	**150th**	63.3±4.6*	48.8±2.7	72.3±3.7***	48.5±4.2	50.9±2.6
**CSA (mm^2)**		1.01±0.07	1.07±0.13	1.05±0.14	1.02±0.09	0.93±0.07
**Number of muscles**		14	15	11	10	9

Numbers below the name of animal group denote the number of animals investigated.

*, ** and *** denotes significant difference compared to aged untrained control at $p<0.05$, 0.01 and 0.001, respectively. [#]The amplitude of the 50th, 100th and 150th tetanus was normalized to the first tetanus. Fatigue was calculated as 100•(1-(normalized tetanus amplitude)). TTP = time to peak; HRT = half relaxation time. [§]Tw = twitch, Te = tetanus. CSA = cross sectional area of the muscle.

Supplementary Table 2. List of monoclonal antibodies used for the detection of different proteins playing important roles in the calcium and selenium homeostasis of skeletal muscle.

**Name of protein**	**Supplier**	**Catalog number**	**Host**	**Dilution**	**Epitope**
**DHPR**	Thermo Scientific	MA3-921	mouse	1/500	purified rabbit dihydropyridine receptor
**RyR1**	I. Marty	Gift from I. Marty	rabbit	1/5000	purified pork ryanodine receptor
**RyR1**	Thermo Scientific	MA3-925	mouse	1/1000	purified chicken pectoral muscle ryanodine receptor
**SERCA1**	Thermo Scientific	MA3-912	mouse	1/1000	amino acid 506-C-terminus of rabbit skeletal muscle ATPase
**Actin**	Santa Cruz	sc-1616	rabbit	1/500	C-terminus of Actin of human origin
**Sepn**	Santa Cruz	sc-98960	rabbit	1/500	amino acid 293-452 in internal region of *SEPN1* of human origin
**α-actinin**	Santa Cruz	sc-166524	mouse	1/500	amino acids 872-891 at the C-terminus of α-actinin of human origin

Supplementary Figure 1

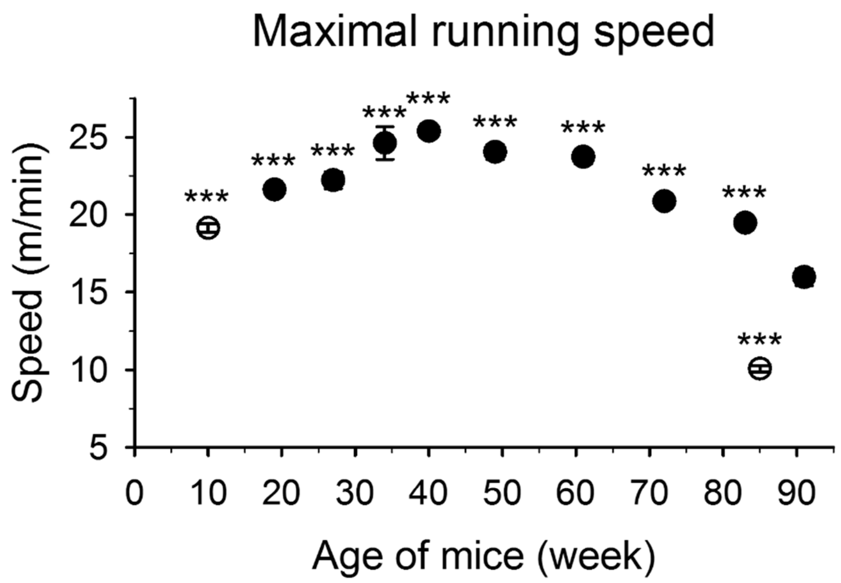

Supplementary Figure 1. Average maximal speed of voluntary running. Average maximal speed of control mice during voluntary running for two weeks in every two month. Empty symbols represent data from measurements on young and aged mice which did not run previously. *** denotes significant difference from the values at 90 week of trained aged animals at p<0.001.

Supplementary Figure 2

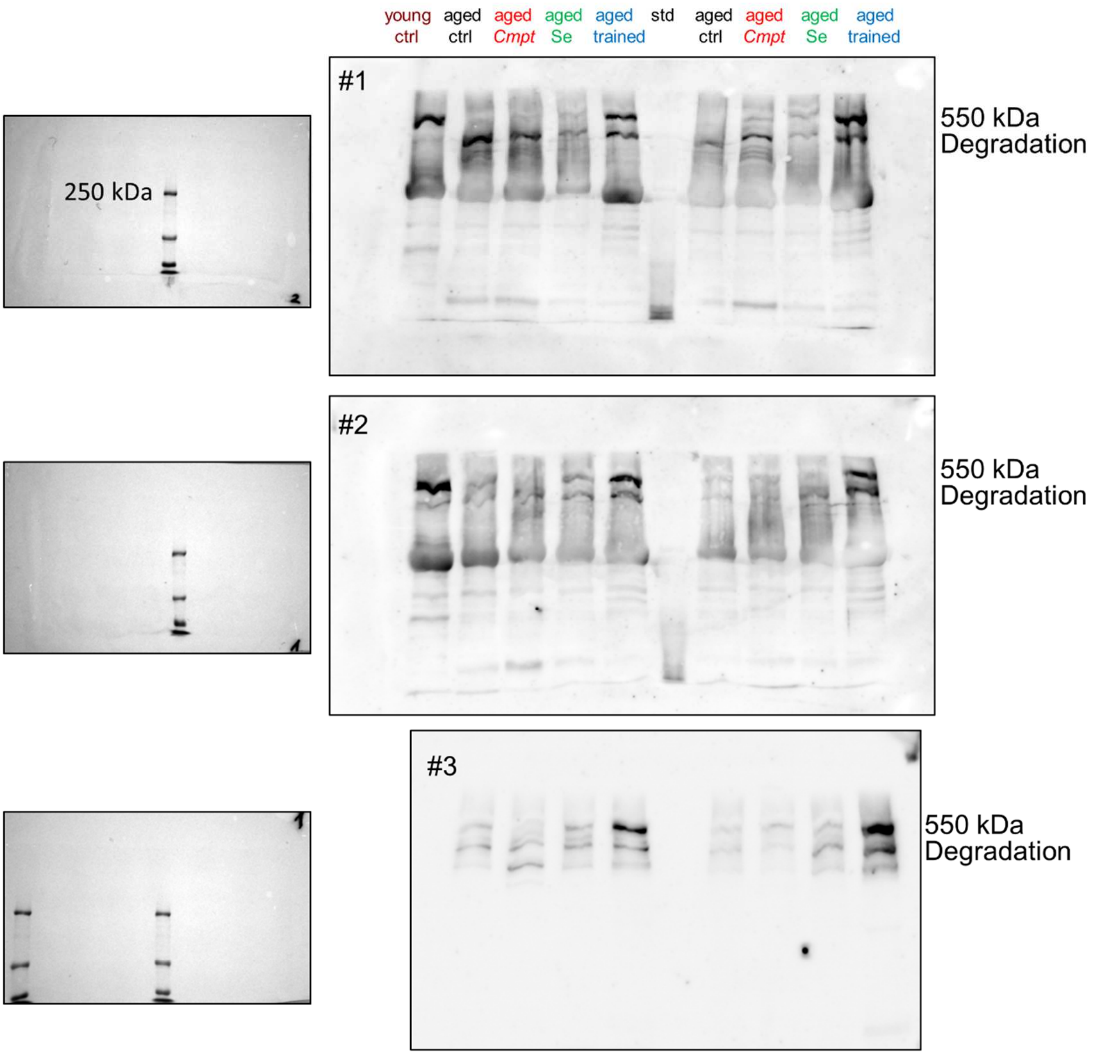

Raw membranes of RyR1 expression in young and different groups of aged mice. Left column shows the standards, detected by transmitted light. Blots #1 and #2 were stained with an antibody from Thermo Scientific, blot #3 was stained with an antibody from I. Marty (see Supplementary Table 2) detected in luminescence mode. Exposition time was defined by gel doc system (automatic calculation) in all cases.

Supplementary Figure 3

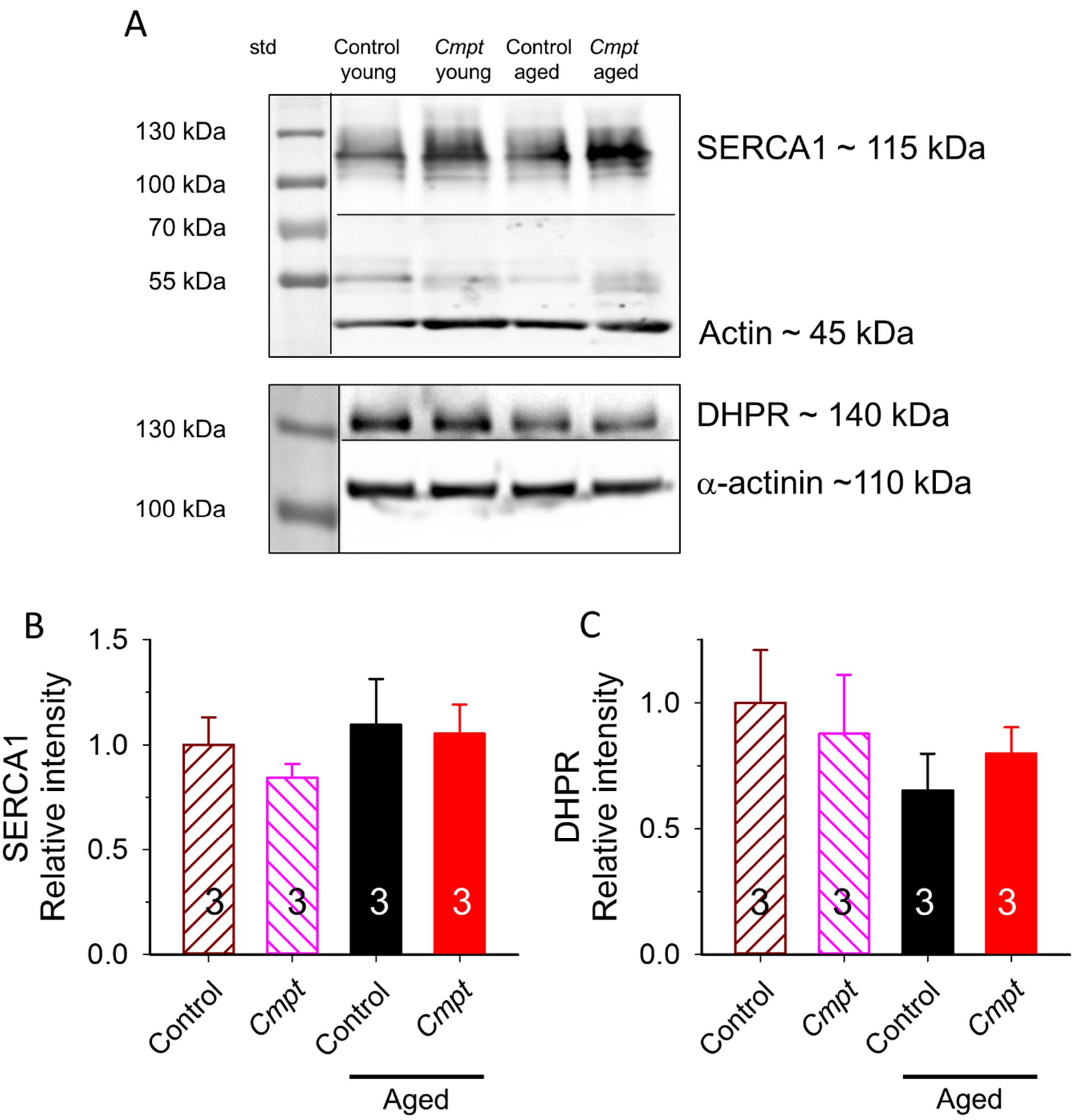

Expression of SERCA1 and DHPR in young and aged muscle.
Representative Western blot images showing the expression of SERCA1, and DHPR in *quadriceps femoris* muscles (A). α-actinin and actin was used as loading control. Averaged expression of SERCA1 (B), and DHPR (C) in muscles from young and aged control, and *Cmpt* mice normalized first to loading control and then to young control. Numbers in bars give the number of animal investigated. Black vertical and horizontal lines indicate the merging border between standard (std) and bands or bands detected with different exposition times (blots with all exposition times are shown in Supplementary Figure 4).

Supplementary Figure 4

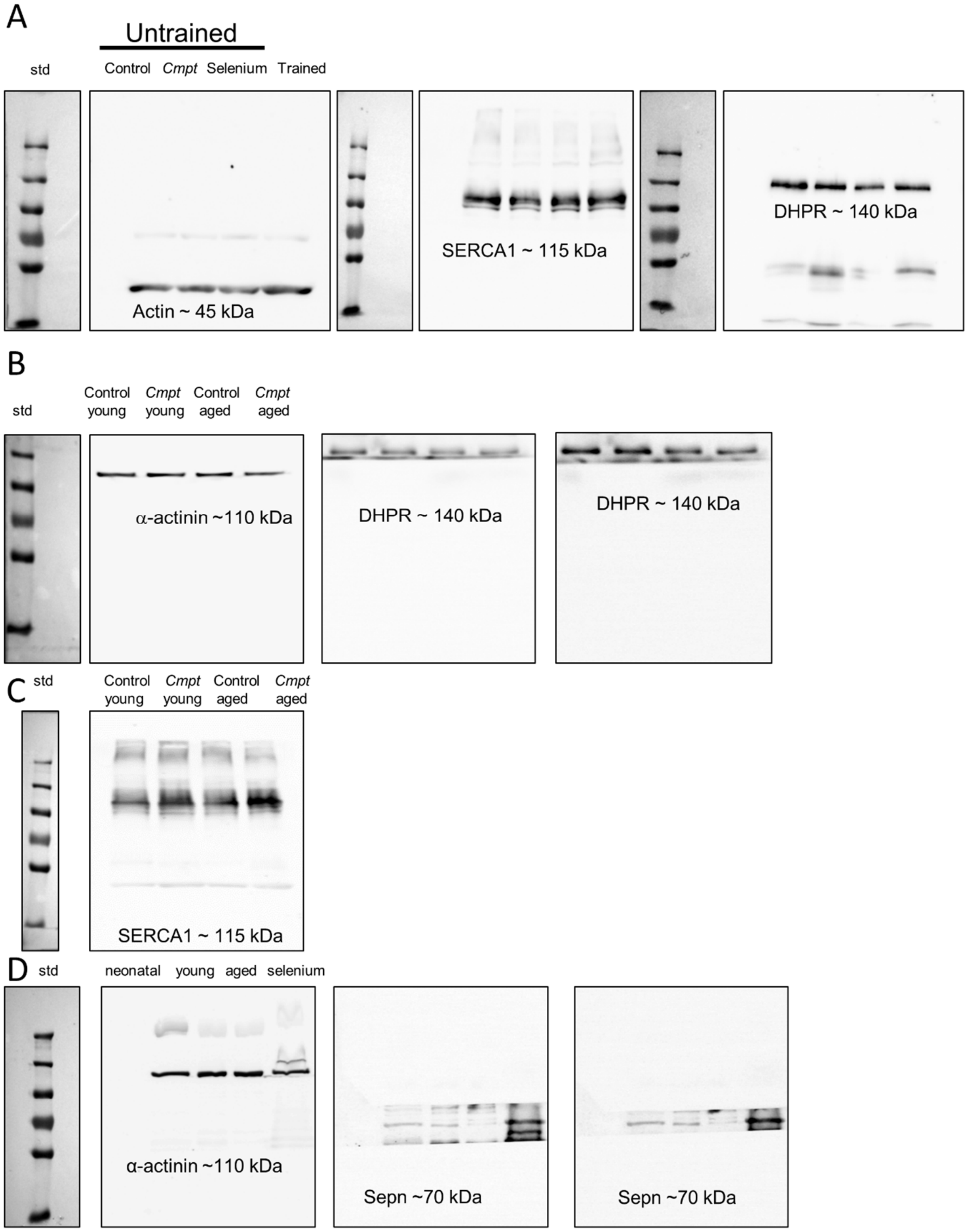

Raw membranes of Western blots presented previously. Panel A corresponds to blots of Figure 6. Panels B & C refer to Supplementary Figure 3. Panel D presents original blots for Figure 7. Standards (35, 55, 70, 100, 130 & 250 kDa) were detected with transmitted light, while the specific bands in luminescence mode. Headings are identical for all blots in panels A, B & D for all blots.

Supplementary Figure 5

Mediation analysis

Model
X: independent parameter
Y: dependent parameter
M: mediator parameter

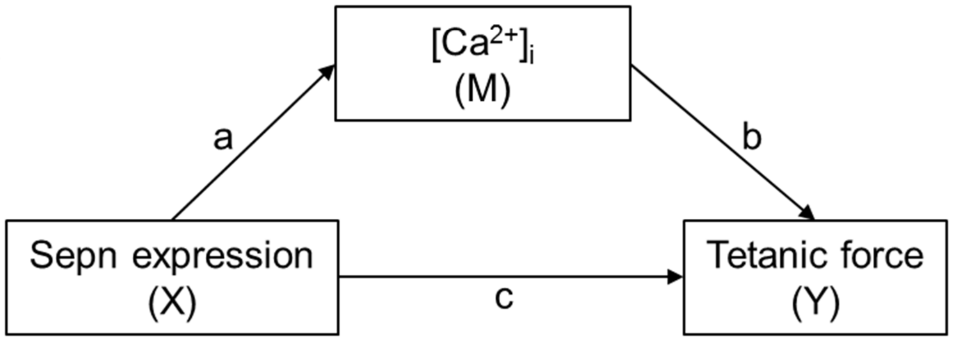

Steps	Analysis	Parameters
Step #1	Conduct a simple regression analysis with X predicting Y to test for path „c" alone, $Y=B_0+B_1 \bullet X+e$	$B_1=199.7$
Step #2	Conduct a simple regression analysis with X predicting M to test for path „a" alone, $M=B_0+B_2 \bullet X+e$	$B_2=1126.7$
Step #3	Conduct a simple regression analysis with M predicting Y to test for path „b" alone, $Y=B_0+B_3 \bullet M+e$	$B_3=0.175$
Step #4	Conduct a multiple regression analysis with X and M predicting Y, $Y=B_0+B_4 \bullet X+B_5 \bullet M+e$	$B_4=202.6$ $B_5=-0.00256$

Method: A causal mediation model was constructed to examine whether the association of selenoprotein with maximal tetanic force was mediated by intracellular Ca^{2+} concentration. The analysis was carried out on data originating from Selenium fed animals.
Note: „e" is representing the error of measurements in the regression equation.
Evaluation: since parameter B_1 and B_4 is almost identical and significantly higher than 0, and B_5 is not significantly higher than 0, $[Ca^{2+}]_i$ has **direct and no mediation** effects on tetanic force.

Statistical analysis of data from Table 1, 2, 3, Supplementary Table 1, Figure 2, 5, 6, 7, and Supplementary Figure 3.

One way ANOVA, single factor, with Bonferroni's multiple comparisons test.

Statistics of data from Table 1

Body weight (g)				
Bonferroni's multiple comparisons test	Mean Diff.	95% CI of diff.	Significant?	Summary
Young control vs. Aged control	-11.48	-16.70 to -6.253	Yes	****
Young control vs. Cmpt	-33.03	-38.04 to -28.03	Yes	****
Young control vs. Selenium fed	-8.908	-14.13 to -3.687	Yes	***
Young control vs. Trained	-7.213	-12.05 to -2.378	Yes	***
Aged control vs. Cmpt	-21.56	-26.94 to -16.18	Yes	****
Aged control vs. Selenium fed	2.567	-3.016 to 8.149	No	ns
Aged control vs. Trained	4.263	-0.9593 to 9.484	Yes	**
Cmpt vs. Selenium fed	24.12	18.74 to 29.50	Yes	****
Cmpt vs. Trained	25.82	20.82 to 30.82	Yes	****
Selenium fed vs. Trained	1.696	-3.526 to 6.918	No	ns
Test details	Mean 1	Mean 2	Mean Diff.	SE of diff.
Young control vs. Aged control	19.73	31.2	-11.48	1.723
Young control vs. Cmpt	19.73	52.76	-33.03	1.652
Young control vs. Selenium fed	19.73	28.63	-8.908	1.723
Young control vs. Trained	19.73	26.94	-7.213	1.596
Aged control vs. Cmpt	31.2	52.76	-21.56	1.775
Aged control vs. Selenium fed	31.2	28.63	2.567	1.842
Aged control vs. Trained	31.2	26.94	4.263	1.723
Cmpt vs. Selenium fed	52.76	28.63	24.12	1.775
Cmpt vs. Trained	52.76	26.94	25.82	1.652
Selenium fed vs. Trained	28.63	26.94	1.696	1.723

Maximal grip force (mN)

Bonferroni's multiple comparisons test	Mean Diff.	95% CI of diff.	Significant?	Summary
Young Control vs. Aged Control	-1.526	-15.12 to 12.07	No	ns
Young Control vs. Cmpt	-40.42	-53.40 to -27.45	Yes	****
Young Control vs. Selenium fed	-6.053	-19.71 to 7.604	No	ns
Young Control vs. Trained	-14.15	-27.31 to -0.9880	Yes	*
Aged Control vs. Cmpt	-38.9	-52.80 to -25.00	Yes	****
Aged Control vs. Selenium fed	-4.527	-19.07 to 10.02	No	ns
Aged Control vs. Trained	-12.62	-26.70 to 1.455	Yes	**
Cmpt vs. Selenium fed	34.37	20.41 to 48.33	Yes	****
Cmpt vs. Trained	26.28	12.80 to 39.75	Yes	****
Selenium fed vs. Trained	-8.095	-22.23 to 6.041	No	ns

Test details	Mean 1	Mean 2	Mean Diff.	SE of diff.
Young Control vs. Aged Control	97.96	99.49	-1.526	4.813
Young Control vs. Cmpt	97.96	138.4	-40.42	4.592
Young Control vs. Selenium fed	97.96	104	-6.053	4.835
Young Control vs. Trained	97.96	112.1	-14.15	4.659
Aged Control vs. Cmpt	99.49	138.4	-38.9	4.921
Aged Control vs. Selenium fed	99.49	104	-4.527	5.148
Aged Control vs. Trained	99.49	112.1	-12.62	4.984
Cmpt vs. Selenium fed	138.4	104	34.37	4.943
Cmpt vs. Trained	138.4	112.1	26.28	4.771
Selenium fed vs. Trained	104	112.1	-8.095	5.004

Normalized maximal grip force to body weight (mN/g)

Bonferroni's multiple comparisons test	Mean Diff.	95% CI of diff.	Significant?	Summary
Young Control vs. Aged Control	1.781	1.369 to 2.193	Yes	****
Young Control vs. Cmpt	2.391	1.998 to 2.785	Yes	****
Young Control vs. Selenium fed	1.368	0.9546 to 1.782	Yes	****
Young Control vs. Trained	0.8312	0.4325 to 1.230	Yes	****
Aged Control vs. Cmpt	0.6105	0.1904 to 1.031	Yes	***
Aged Control vs. Selenium fed	-0.4126	-0.8521 to 0.0268	Yes	**
Aged Control vs. Trained	-0.9497	-1.375 to -0.5243	Yes	****
Cmpt vs. Selenium fed	-1.023	-1.445 to -0.6013	Yes	****
Cmpt vs. Trained	-1.56	-1.967 to -1.153	Yes	****
Selenium fed vs. Trained	-0.5371	-0.9643 to -0.1099	Yes	**

Test details	Mean 1	Mean 2	Mean Diff.	SE of diff.
Young Control vs. Aged Control	5.001	3.22	1.781	0.1458
Young Control vs. Cmpt	5.001	2.61	2.391	0.1392
Young Control vs. Selenium fed	5.001	3.633	1.368	0.1465
Young Control vs. Trained	5.001	4.17	0.8312	0.1412
Aged Control vs. Cmpt	3.22	2.61	0.6105	0.1487
Aged Control vs. Selenium fed	3.22	3.633	-0.4126	0.1556
Aged Control vs. Trained	3.22	4.17	-0.9497	0.1506
Cmpt vs. Selenium fed	2.61	3.633	-1.023	0.1494
Cmpt vs. Trained	2.61	4.17	-1.56	0.1442
Selenium fed vs. Trained	3.633	4.17	-0.5371	0.1512

Statistics of data from Table 2 and Figure 2.

EDL Twitch Peak force (mN/mm2)

Bonferroni's multiple comparisons test	Mean Diff.	95% CI of diff.	Significant?	Summary
Young Control vs. Aged Control	0.669	-0.1548 to 1.493	Yes	*
Young Control vs. Cmpt	1.286	0.3208 to 2.250	Yes	**
Young Control vs. Selenium fed	0.04353	-0.8221 to 0.9092	No	ns
Young Control vs. Trained	-0.1742	-1.067 to 0.7182	No	ns
Aged Control vs. Cmpt	0.6166	-0.3622 to 1.595	Yes	*
Aged Control vs. Selenium fed	-0.6255	-1.507 to 0.2557	Yes	*
Aged Control vs. Trained	-0.8433	-1.751 to 0.06427	Yes	*
Cmpt vs. Selenium fed	-1.242	-2.256 to -0.2279	Yes	**
Cmpt vs. Trained	-1.46	-2.497 to -0.4227	Yes	**
Selenium fed vs. Trained	-0.2177	-1.163 to 0.7279	No	ns
**Test details**	**Mean 1**	**Mean 2**	**Mean Diff.**	**SE of diff.**
Young Control vs. Aged Control	2.377	1.708	0.669	0.2794
Young Control vs. Cmpt	2.377	1.091	1.286	0.3272
Young Control vs. Selenium fed	2.377	2.333	0.04353	0.2936
Young Control vs. Trained	2.377	2.551	-0.1742	0.3026
Aged Control vs. Cmpt	1.708	1.091	0.6166	0.3319
Aged Control vs. Selenium fed	1.708	2.333	-0.6255	0.2988
Aged Control vs. Trained	1.708	2.551	-0.8433	0.3078
Cmpt vs. Selenium fed	1.091	2.333	-1.242	0.3439
Cmpt vs. Trained	1.091	2.551	-1.46	0.3517
Selenium fed vs. Trained	2.333	2.551	-0.2177	0.3207

EDL Twitch TTP (ms)

Bonferroni's multiple comparisons test	Mean Diff.	95% CI of diff.	Significant?	Summary
Young Control vs. Aged Control	2.265	-1.220 to 5.750	No	ns
Young Control vs. Cmpt	4.27	0.3119 to 8.228	Yes	*
Young Control vs. Selenium fed	1.975	-1.983 to 5.933	No	ns
Young Control vs. Trained	1.918	-2.170 to 6.006	No	ns
Aged Control vs. Cmpt	2.005	-1.903 to 5.913	No	ns
Aged Control vs. Selenium fed	-0.29	-4.198 to 3.618	No	ns
Aged Control vs. Trained	-0.3472	-4.387 to 3.693	No	ns
Cmpt vs. Selenium fed	-2.295	-6.631 to 2.041	No	ns
Cmpt vs. Trained	-2.352	-6.807 to 2.103	No	ns
Selenium fed vs. Trained	-0.05722	-4.512 to 4.398	No	ns
**Test details**	**Mean 1**	**Mean 2**	**Mean Diff.**	**SE of diff.**
Young Control vs. Aged Control	34.64	32.38	2.265	1.191
Young Control vs. Cmpt	34.64	30.37	4.27	1.353
Young Control vs. Selenium fed	34.64	32.67	1.975	1.353
Young Control vs. Trained	34.64	32.72	1.918	1.398
Aged Control vs. Cmpt	32.38	30.37	2.005	1.336
Aged Control vs. Selenium fed	32.38	32.67	-0.29	1.336
Aged Control vs. Trained	32.38	32.72	-0.3472	1.381
Cmpt vs. Selenium fed	30.37	32.67	-2.295	1.483
Cmpt vs. Trained	30.37	32.72	-2.352	1.523
Selenium fed vs. Trained	32.67	32.72	-0.05722	1.523

EDL Twitch HRT (ms)

Bonferroni's multiple comparisons test	Mean Diff.	95% CI of diff.	Significant?	Summary
Young Control vs. Aged Control	-1.941	-6.656 to 2.774	No	ns
Young Control vs. Cmpt	1.019	-4.337 to 6.375	No	ns
Young Control vs. Selenium fed	-0.93	-6.286 to 4.426	No	ns
Young Control vs. Trained	1.534	-3.997 to 7.066	No	ns
Aged Control vs. Cmpt	2.96	-2.328 to 8.248	No	ns
Aged Control vs. Selenium fed	1.011	-4.277 to 6.300	No	ns
Aged Control vs. Trained	3.476	-1.990 to 8.942	Yes	*
Cmpt vs. Selenium fed	-1.949	-7.816 to 3.918	No	ns
Cmpt vs. Trained	0.5157	-5.512 to 6.543	No	ns
Selenium fed vs. Trained	2.464	-3.563 to 8.492	No	ns

Test details	Mean 1	Mean 2	Mean Diff.	SE of diff.
Young Control vs. Aged Control	28.99	30.93	-1.941	1.612
Young Control vs. Cmpt	28.99	27.97	1.019	1.831
Young Control vs. Selenium fed	28.99	29.92	-0.93	1.831
Young Control vs. Trained	28.99	27.46	1.534	1.891
Aged Control vs. Cmpt	30.93	27.97	2.96	1.808
Aged Control vs. Selenium fed	30.93	29.92	1.011	1.808
Aged Control vs. Trained	30.93	27.46	3.476	1.869
Cmpt vs. Selenium fed	27.97	29.92	-1.949	2.006
Cmpt vs. Trained	27.97	27.46	0.5157	2.061
Selenium fed vs. Trained	29.92	27.46	2.464	2.061

EDL Twitch Duration (ms)

Bonferroni's multiple comparisons test	Mean Diff.	95% CI of diff.	Significant?	Summary
Young Control vs. Aged Control	31.12	-62.46 to 124.7	No	ns
Young Control vs. Cmpt	80.05	-29.74 to 189.8	No	ns
Young Control vs. Selenium fed	37.25	-69.05 to 143.5	No	ns
Young Control vs. Trained	84.01	-35.17 to 203.2	No	ns
Aged Control vs. Cmpt	48.93	-59.57 to 157.4	No	ns
Aged Control vs. Selenium fed	6.128	-98.83 to 111.1	No	ns
Aged Control vs. Trained	52.89	-65.10 to 170.9	No	ns
Cmpt vs. Selenium fed	-42.8	-162.4 to 76.84	No	ns
Cmpt vs. Trained	3.968	-127.3 to 135.2	No	ns
Selenium fed vs. Trained	46.77	-81.55 to 175.1	No	ns

Test details	Mean 1	Mean 2	Mean Diff.	SE of diff.
Young Control vs. Aged Control	216.5	185.3	31.12	31.92
Young Control vs. Cmpt	216.5	136.4	80.05	37.45
Young Control vs. Selenium fed	216.5	179.2	37.25	36.26
Young Control vs. Trained	216.5	132.4	84.01	40.65
Aged Control vs. Cmpt	185.3	136.4	48.93	37.01
Aged Control vs. Selenium fed	185.3	179.2	6.128	35.8
Aged Control vs. Trained	185.3	132.4	52.89	40.25
Cmpt vs. Selenium fed	136.4	179.2	-42.8	40.81
Cmpt vs. Trained	136.4	132.4	3.968	44.76
Selenium fed vs. Trained	179.2	132.4	46.77	43.77

EDL Twitch Twitch/Tetanus				
Bonferroni's multiple comparisons test	Mean Diff.	95% CI of diff.	Significant?	Summary
Young Control vs. Aged Control	-0.02029	-0.1332 to 0.09263	No	ns
Young Control vs. Cmpt	-0.07484	-0.2161 to 0.06644	No	ns
Young Control vs. Selenium fed	-0.01123	-0.1242 to 0.1017	No	ns
Young Control vs. Trained	-0.01332	-0.1546 to 0.1280	No	ns
Aged Control vs. Cmpt	-0.05455	-0.2016 to 0.09250	No	ns
Aged Control vs. Selenium fed	0.009055	-0.1110 to 0.1291	No	ns
Aged Control vs. Trained	0.006975	-0.1401 to 0.1540	No	ns
Cmpt vs. Selenium fed	0.0636	-0.08344 to 0.2107	No	ns
Cmpt vs. Trained	0.06152	-0.1083 to 0.2313	No	ns
Selenium fed vs. Trained	-0.002081	-0.1491 to 0.1450	No	ns
Test details	Mean 1	Mean 2	Mean Diff.	SE of diff.
Young Control vs. Aged Control	0.2026	0.2229	-0.02029	0.03789
Young Control vs. Cmpt	0.2026	0.2774	-0.07484	0.0474
Young Control vs. Selenium fed	0.2026	0.2138	-0.01123	0.03789
Young Control vs. Trained	0.2026	0.2159	-0.01332	0.0474
Aged Control vs. Cmpt	0.2229	0.2774	-0.05455	0.04934
Aged Control vs. Selenium fed	0.2229	0.2138	0.009055	0.04029
Aged Control vs. Trained	0.2229	0.2159	0.006975	0.04934
Cmpt vs. Selenium fed	0.2774	0.2138	0.0636	0.04934
Cmpt vs. Trained	0.2774	0.2159	0.06152	0.05697
Selenium fed vs. Trained	0.2138	0.2159	-0.002081	0.04934

EDL Twitch CSA (mm2)				
Bonferroni's multiple comparisons test	Mean Diff.	95% CI of diff.	Significant?	Summary
Young Control vs. Aged Control	-0.1863	-0.5563 to 0.1837	Yes	*
Young Control vs. Cmpt	-0.8703	-1.292 to -0.4484	Yes	****
Young Control vs. Selenium fed	0.1208	-0.3011 to 0.5426	No	ns
Young Control vs. Trained	0.2245	-0.2116 to 0.6605	No	ns
Aged Control vs. Cmpt	-0.684	-1.106 to -0.2621	Yes	***
Aged Control vs. Selenium fed	0.3071	-0.1148 to 0.7289	Yes	*
Aged Control vs. Trained	0.4108	-0.02529 to 0.8468	Yes	**
Cmpt vs. Selenium fed	0.9911	0.5230 to 1.459	Yes	****
Cmpt vs. Trained	1.095	0.6139 to 1.576	Yes	****
Selenium fed vs. Trained	0.1037	-0.3772 to 0.5846	No	ns
Test details	Mean 1	Mean 2	Mean Diff.	SE of diff.
Young Control vs. Aged Control	1.029	1.215	-0.1863	0.1266
Young Control vs. Cmpt	1.029	1.899	-0.8703	0.1444
Young Control vs. Selenium fed	1.029	0.9083	0.1208	0.1444
Young Control vs. Trained	1.029	0.8046	0.2245	0.1492
Aged Control vs. Cmpt	1.215	1.899	-0.684	0.1444
Aged Control vs. Selenium fed	1.215	0.9083	0.3071	0.1444
Aged Control vs. Trained	1.215	0.8046	0.4108	0.1492
Cmpt vs. Selenium fed	1.899	0.9083	0.9911	0.1601
Cmpt vs. Trained	1.899	0.8046	1.095	0.1645
Selenium fed vs. Trained	0.9083	0.8046	0.1037	0.1645

EDL Tetanus Peak force (mN/mm2)				
Bonferroni's multiple comparisons test	Mean Diff.	95% CI of diff.	Significant?	Summary
Young Control vs. Aged Control	3.15	-0.2085 to 6.509	Yes	**
Young Control vs. Cmpt	5.884	1.579 to 10.19	Yes	**
Young Control vs. Selenium fed	-0.05907	-3.889 to 3.771	No	ns
Young Control vs. Trained	-2.502	-6.461 to 1.456	No	ns
Aged Control vs. Cmpt	2.734	-1.571 to 7.039	Yes	*
Aged Control vs. Selenium fed	-3.209	-7.039 to 0.6203	Yes	**
Aged Control vs. Trained	-5.652	-9.611 to -1.694	Yes	***
Cmpt vs. Selenium fed	-5.943	-10.62 to -1.261	Yes	**
Cmpt vs. Trained	-8.386	-13.17 to -3.598	Yes	****
Selenium fed vs. Trained	-2.443	-6.808 to 1.922	No	ns
Test details	Mean 1	Mean 2	Mean Diff.	SE of diff.
Young Control vs. Aged Control	10.83	7.682	3.15	1.147
Young Control vs. Cmpt	10.83	4.948	5.884	1.47
Young Control vs. Selenium fed	10.83	10.89	-0.05907	1.307
Young Control vs. Trained	10.83	13.33	-2.502	1.351
Aged Control vs. Cmpt	7.682	4.948	2.734	1.47
Aged Control vs. Selenium fed	7.682	10.89	-3.209	1.307
Aged Control vs. Trained	7.682	13.33	-5.652	1.351
Cmpt vs. Selenium fed	4.948	10.89	-5.943	1.598
Cmpt vs. Trained	4.948	13.33	-8.386	1.634
Selenium fed vs. Trained	10.89	13.33	-2.443	1.49

EDL Tetanus TTP (ms)				
Bonferroni's multiple comparisons test	Mean Diff.	95% CI of diff.	Significant?	Summary
Young Control vs. Aged Control	4.381	-15.85 to 24.61	No	ns
Young Control vs. Cmpt	8.472	-14.59 to 31.54	No	ns
Young Control vs. Selenium fed	-16.06	-39.13 to 7.002	No	ns
Young Control vs. Trained	-15.01	-38.85 to 8.832	No	ns
Aged Control vs. Cmpt	4.091	-18.97 to 27.16	No	ns
Aged Control vs. Selenium fed	-20.44	-43.51 to 2.621	Yes	*
Aged Control vs. Trained	-19.39	-43.23 to 4.451	No	ns
Cmpt vs. Selenium fed	-24.54	-50.12 to 1.053	No	ns
Cmpt vs. Trained	-23.48	-49.77 to 2.809	No	ns
Selenium fed vs. Trained	1.054	-25.24 to 27.34	No	ns
Test details	Mean 1	Mean 2	Mean Diff.	SE of diff.
Young Control vs. Aged Control	173.2	168.8	4.381	6.922
Young Control vs. Cmpt	173.2	164.7	8.472	7.892
Young Control vs. Selenium fed	173.2	189.3	-16.06	7.892
Young Control vs. Trained	173.2	188.2	-15.01	8.158
Aged Control vs. Cmpt	168.8	164.7	4.091	7.892
Aged Control vs. Selenium fed	168.8	189.3	-20.44	7.892
Aged Control vs. Trained	168.8	188.2	-19.39	8.158
Cmpt vs. Selenium fed	164.7	189.3	-24.54	8.756
Cmpt vs. Trained	164.7	188.2	-23.48	8.996
Selenium fed vs. Trained	189.3	188.2	1.054	8.996

EDL Tetanus HRT (ms)

Bonferroni's multiple comparisons test	Mean Diff.	95% CI of diff.	Significant?	Summary
Young Control vs. Aged Control	-2.5	-23.13 to 18.13	No	ns
Young Control vs. Cmpt	-1.719	-25.24 to 21.80	No	ns
Young Control vs. Selenium fed	8.661	-14.86 to 32.18	No	ns
Young Control vs. Trained	9.935	-14.38 to 34.25	No	ns
Aged Control vs. Cmpt	0.7806	-22.74 to 24.30	No	ns
Aged Control vs. Selenium fed	11.16	-12.36 to 34.68	No	ns
Aged Control vs. Trained	12.44	-11.88 to 36.75	No	ns
Cmpt vs. Selenium fed	10.38	-15.71 to 36.47	No	ns
Cmpt vs. Trained	11.65	-15.15 to 38.46	No	ns
Selenium fed vs. Trained	1.274	-25.53 to 28.08	No	ns
**Test details**	**Mean 1**	**Mean 2**	**Mean Diff.**	**SE of diff.**
Young Control vs. Aged Control	72.84	75.34	-2.5	7.058
Young Control vs. Cmpt	72.84	74.56	-1.719	8.048
Young Control vs. Selenium fed	72.84	64.18	8.661	8.048
Young Control vs. Trained	72.84	62.91	9.935	8.318
Aged Control vs. Cmpt	75.34	74.56	0.7806	8.048
Aged Control vs. Selenium fed	75.34	64.18	11.16	8.048
Aged Control vs. Trained	75.34	62.91	12.44	8.318
Cmpt vs. Selenium fed	74.56	64.18	10.38	8.928
Cmpt vs. Trained	74.56	62.91	11.65	9.173
Selenium fed vs. Trained	64.18	62.91	1.274	9.173

EDL Tetanus Duration (ms)

Bonferroni's multiple comparisons test	Mean Diff.	95% CI of diff.	Significant?	Summary
Young Control vs. Aged Control	6.434	-20.20 to 33.06	No	ns
Young Control vs. Cmpt	14.02	-16.34 to 44.39	No	ns
Young Control vs. Selenium fed	-25.23	-55.59 to 5.136	No	ns
Young Control vs. Trained	-14.1	-45.49 to 17.28	No	ns
Aged Control vs. Cmpt	7.589	-22.77 to 37.95	No	ns
Aged Control vs. Selenium fed	-31.66	-62.02 to -1.298	Yes	*
Aged Control vs. Trained	-20.54	-51.92 to 10.85	No	ns
Cmpt vs. Selenium fed	-39.25	-72.94 to -5.565	Yes	*
Cmpt vs. Trained	-28.13	-62.73 to 6.483	No	ns
Selenium fed vs. Trained	11.12	-23.48 to 45.73	No	ns
**Test details**	**Mean 1**	**Mean 2**	**Mean Diff.**	**SE of diff.**
Young Control vs. Aged Control	336.1	329.7	6.434	9.112
Young Control vs. Cmpt	336.1	322.1	14.02	10.39
Young Control vs. Selenium fed	336.1	361.3	-25.23	10.39
Young Control vs. Trained	336.1	350.2	-14.1	10.74
Aged Control vs. Cmpt	329.7	322.1	7.589	10.39
Aged Control vs. Selenium fed	329.7	361.3	-31.66	10.39
Aged Control vs. Trained	329.7	350.2	-20.54	10.74
Cmpt vs. Selenium fed	322.1	361.3	-39.25	11.53
Cmpt vs. Trained	322.1	350.2	-28.13	11.84
Selenium fed vs. Trained	361.3	350.2	11.12	11.84

EDL Tetanus 50th Fatigue

Bonferroni's multiple comparisons test	Mean Diff.	95% CI of diff.	Significant?	Summary
Young Control vs. Aged Control	-0.008218	-0.1010 to 0.08453	No	ns
Young Control vs. Cmpt	-0.08483	-0.2005 to 0.03084	No	ns
Young Control vs. Selenium fed	0.09303	-0.007146 to 0.1932	No	ns
Young Control vs. Trained	0.1016	-0.008801 to 0.2120	No	ns
Aged Control vs. Cmpt	-0.07662	-0.1859 to 0.03269	No	ns
Aged Control vs. Selenium fed	0.1012	0.008503 to 0.1940	Yes	***
Aged Control vs. Trained	0.1098	0.006113 to 0.2135	Yes	**
Cmpt vs. Selenium fed	0.1779	0.06219 to 0.2935	Yes	***
Cmpt vs. Trained	0.1864	0.06180 to 0.3110	Yes	***
Selenium fed vs. Trained	0.008557	-0.1018 to 0.1189	No	ns

Test details	Mean 1	Mean 2	Mean Diff.	SE of diff.
Young Control vs. Aged Control	0.3164	0.3246	-0.008218	0.0313
Young Control vs. Cmpt	0.3164	0.4012	-0.08483	0.03904
Young Control vs. Selenium fed	0.3164	0.2234	0.09303	0.03381
Young Control vs. Trained	0.3164	0.2148	0.1016	0.03726
Aged Control vs. Cmpt	0.3246	0.4012	-0.07662	0.03689
Aged Control vs. Selenium fed	0.3246	0.2234	0.1012	0.0313
Aged Control vs. Trained	0.3246	0.2148	0.1098	0.035
Cmpt vs. Selenium fed	0.4012	0.2234	0.1779	0.03904
Cmpt vs. Trained	0.4012	0.2148	0.1864	0.04206
Selenium fed vs. Trained	0.2234	0.2148	0.008557	0.03726

EDL Tetanus 100th Fatigue

Bonferroni's multiple comparisons test	Mean Diff.	95% CI of diff.	Significant?	Summary
Young Control vs. Aged Control	0.05707	-0.05644 to 0.1706	No	ns
Young Control vs. Cmpt	-0.07248	-0.2125 to 0.06754	No	ns
Young Control vs. Selenium fed	0.199	0.07692 to 0.3211	Yes	***
Young Control vs. Trained	0.1734	0.03951 to 0.3073	Yes	**
Aged Control vs. Cmpt	-0.1295	-0.2592 to 8.603e-005	Yes	*
Aged Control vs. Selenium fed	0.1419	0.03192 to 0.2519	Yes	***
Aged Control vs. Trained	0.1163	-0.006658 to 0.2393	Yes	**
Cmpt vs. Selenium fed	0.2715	0.1343 to 0.4087	Yes	****
Cmpt vs. Trained	0.2459	0.09807 to 0.3937	Yes	***
Selenium fed vs. Trained	-0.0256	-0.1565 to 0.1053	No	ns

Test details	Mean 1	Mean 2	Mean Diff.	SE of diff.
Young Control vs. Aged Control	0.6043	0.5472	0.05707	0.03826
Young Control vs. Cmpt	0.6043	0.6768	-0.07248	0.04719
Young Control vs. Selenium fed	0.6043	0.4053	0.199	0.04114
Young Control vs. Trained	0.6043	0.4309	0.1734	0.04513
Aged Control vs. Cmpt	0.5472	0.6768	-0.1295	0.04369
Aged Control vs. Selenium fed	0.5472	0.4053	0.1419	0.03707
Aged Control vs. Trained	0.5472	0.4309	0.1163	0.04145
Cmpt vs. Selenium fed	0.6768	0.4053	0.2715	0.04624
Cmpt vs. Trained	0.6768	0.4309	0.2459	0.04982
Selenium fed vs. Trained	0.4053	0.4309	-0.0256	0.04413

EDL Tetanus 150th Fatigue				
**Bonferroni's multiple comparisons test**	**Mean Diff.**	**95% CI of diff.**	**Significant?**	**Summary**
**Young Control vs. Aged Control**	-0.005357	-0.1313 to 0.1206	No	ns
**Young Control vs. Cmpt**	-0.1239	-0.2793 to 0.03139	No	ns
**Young Control vs. Selenium fed**	0.1854	0.05000 to 0.3208	Yes	**
**Young Control vs. Trained**	0.1911	0.03580 to 0.3465	Yes	**
**Aged Control vs. Cmpt**	-0.1186	-0.2624 to 0.02522	Yes	*
**Aged Control vs. Selenium fed**	0.1908	0.06874 to 0.3128	Yes	***
**Aged Control vs. Trained**	0.1965	0.05268 to 0.3403	Yes	***
**Cmpt vs. Selenium fed**	0.3094	0.1572 to 0.4615	Yes	****
**Cmpt vs. Trained**	0.3151	0.1449 to 0.4852	Yes	****
**Selenium fed vs. Trained**	0.005718	-0.1465 to 0.1579	No	ns
**Test details**	**Mean 1**	**Mean 2**	**Mean Diff.**	**SE of diff.**
**Young Control vs. Aged Control**	0.6762	0.6815	-0.005357	0.04238
**Young Control vs. Cmpt**	0.6762	0.8001	-0.1239	0.05228
**Young Control vs. Selenium fed**	0.6762	0.4908	0.1854	0.04558
**Young Control vs. Trained**	0.6762	0.485	0.1911	0.05228
**Aged Control vs. Cmpt**	0.6815	0.8001	-0.1186	0.0484
**Aged Control vs. Selenium fed**	0.6815	0.4908	0.1908	0.04107
**Aged Control vs. Trained**	0.6815	0.485	0.1965	0.0484
**Cmpt vs. Selenium fed**	0.8001	0.4908	0.3094	0.05122
**Cmpt vs. Trained**	0.8001	0.485	0.3151	0.05727
**Selenium fed vs. Trained**	0.4908	0.485	0.005718	0.05122

Statistics of data from Supplementary Table 1.

Soleus Twitch Peak force (mN/mm2)

Bonferroni's multiple comparisons test	Mean Diff.	95% CI of diff.	Significant?	Summary
Young Control vs. Aged Control	0.0346	-1.160 to 1.229	No	ns
Young Control vs. Cmpt	0.2337	-1.091 to 1.558	No	ns
Young Control vs. Selenium fed	-0.005324	-1.289 to 1.278	No	ns
Young Control vs. Trained	0.04952	-1.275 to 1.374	No	ns
Aged Control vs. Cmpt	0.1991	-1.145 to 1.544	No	ns
Aged Control vs. Selenium fed	-0.03992	-1.344 to 1.264	No	ns
Aged Control vs. Trained	0.01492	-1.329 to 1.359	No	ns
Cmpt vs. Selenium fed	-0.2391	-1.664 to 1.185	No	ns
Cmpt vs. Trained	-0.1842	-1.646 to 1.277	No	ns
Selenium fed vs. Trained	0.05484	-1.370 to 1.479	No	ns
**Test details**	**Mean 1**	**Mean 2**	**Mean Diff.**	**SE of diff.**
Young Control vs. Aged Control	2.06	2.026	0.0346	0.4066
Young Control vs. Cmpt	2.06	1.827	0.2337	0.451
Young Control vs. Selenium fed	2.06	2.066	-0.005324	0.4371
Young Control vs. Trained	2.06	2.011	0.04952	0.451
Aged Control vs. Cmpt	2.026	1.827	0.1991	0.4577
Aged Control vs. Selenium fed	2.026	2.066	-0.03992	0.444
Aged Control vs. Trained	2.026	2.011	0.01492	0.4577
Cmpt vs. Selenium fed	1.827	2.066	-0.2391	0.485
Cmpt vs. Trained	1.827	2.011	-0.1842	0.4976
Selenium fed vs. Trained	2.066	2.011	0.05484	0.485

Soleus Twitch TTP (ms)

Bonferroni's multiple comparisons test	Mean Diff.	95% CI of diff.	Significant?	Summary
Young Control vs. Aged Control	5.804	-10.24 to 21.85	No	ns
Young Control vs. Cmpt	2.306	-14.48 to 19.09	No	ns
Young Control vs. Selenium fed	-10.65	-27.90 to 6.605	No	ns
Young Control vs. Trained	-27.17	-44.97 to -9.372	Yes	***
Aged Control vs. Cmpt	-3.498	-20.57 to 13.57	No	ns
Aged Control vs. Selenium fed	-16.45	-33.97 to 1.075	Yes	**
Aged Control vs. Trained	-32.98	-51.04 to -14.91	Yes	****
Cmpt vs. Selenium fed	-12.95	-31.15 to 5.253	No	ns
Cmpt vs. Trained	-29.48	-48.20 to -10.75	Yes	***
Selenium fed vs. Trained	-16.53	-35.67 to 2.616	No	ns
**Test details**	**Mean 1**	**Mean 2**	**Mean Diff.**	**SE of diff.**
Young Control vs. Aged Control	70.5	64.7	5.804	5.474
Young Control vs. Cmpt	70.5	68.19	2.306	5.726
Young Control vs. Selenium fed	70.5	81.15	-10.65	5.884
Young Control vs. Trained	70.5	97.67	-27.17	6.072
Aged Control vs. Cmpt	64.7	68.19	-3.498	5.822
Aged Control vs. Selenium fed	64.7	81.15	-16.45	5.977
Aged Control vs. Trained	64.7	97.67	-32.98	6.162
Cmpt vs. Selenium fed	68.19	81.15	-12.95	6.209
Cmpt vs. Trained	68.19	97.67	-29.48	6.387
Selenium fed vs. Trained	81.15	97.67	-16.53	6.529

**Soleus Twitch HRT (ms)**				
**Bonferroni's multiple comparisons test**	**Mean Diff.**	**95% CI of diff.**	**Significant?**	**Summary**
**Young Control vs. Aged Control**	3.765	-15.13 to 22.66	No	ns
**Young Control vs. Cmpt**	1.72	-18.04 to 21.49	No	ns
**Young Control vs. Selenium fed**	-6.296	-26.61 to 14.01	No	ns
**Young Control vs. Trained**	-19	-39.96 to 1.957	No	ns
**Aged Control vs. Cmpt**	-2.045	-22.14 to 18.05	No	ns
**Aged Control vs. Selenium fed**	-10.06	-30.70 to 10.57	No	ns
**Aged Control vs. Trained**	-22.77	-44.04 to -1.495	Yes	*
**Cmpt vs. Selenium fed**	-8.017	-29.45 to 13.42	No	ns
**Cmpt vs. Trained**	-20.72	-42.77 to 1.327	No	ns
**Selenium fed vs. Trained**	-12.71	-35.25 to 9.834	No	ns
**Test details**	**Mean 1**	**Mean 2**	**Mean Diff.**	**SE of diff.**
**Young Control vs. Aged Control**	67.1	63.34	3.765	6.445
**Young Control vs. Cmpt**	67.1	65.38	1.72	6.742
**Young Control vs. Selenium fed**	67.1	73.4	-6.296	6.928
**Young Control vs. Trained**	67.1	86.11	-19	7.149
**Aged Control vs. Cmpt**	63.34	65.38	-2.045	6.855
**Aged Control vs. Selenium fed**	63.34	73.4	-10.06	7.038
**Aged Control vs. Trained**	63.34	86.11	-22.77	7.256
**Cmpt vs. Selenium fed**	65.38	73.4	-8.017	7.311
**Cmpt vs. Trained**	65.38	86.11	-20.72	7.521
**Selenium fed vs. Trained**	73.4	86.11	-12.71	7.688

**Soleus Twitch Duration (ms)**				
**Bonferroni's multiple comparisons test**	**Mean Diff.**	**95% CI of diff.**	**Significant?**	**Summary**
**Young Control vs. Aged Control**	24.21	-53.92 to 102.3	No	ns
**Young Control vs. Cmpt**	37.96	-43.76 to 119.7	No	ns
**Young Control vs. Selenium fed**	4.362	-79.61 to 88.34	No	ns
**Young Control vs. Trained**	-53.75	-140.4 to 32.91	No	ns
**Aged Control vs. Cmpt**	13.76	-69.33 to 96.85	No	ns
**Aged Control vs. Selenium fed**	-19.84	-105.2 to 65.47	No	ns
**Aged Control vs. Trained**	-77.95	-165.9 to 9.999	Yes	*
**Cmpt vs. Selenium fed**	-33.6	-122.2 to 55.02	No	ns
**Cmpt vs. Trained**	-91.71	-182.9 to -0.5460	Yes	*
**Selenium fed vs. Trained**	-58.11	-151.3 to 35.08	No	ns
**Test details**	**Mean 1**	**Mean 2**	**Mean Diff.**	**SE of diff.**
**Young Control vs. Aged Control**	311.1	286.9	24.21	26.65
**Young Control vs. Cmpt**	311.1	273.1	37.96	27.87
**Young Control vs. Selenium fed**	311.1	306.7	4.362	28.64
**Young Control vs. Trained**	311.1	364.8	-53.75	29.56
**Aged Control vs. Cmpt**	286.9	273.1	13.76	28.34
**Aged Control vs. Selenium fed**	286.9	306.7	-19.84	29.1
**Aged Control vs. Trained**	286.9	364.8	-77.95	30
**Cmpt vs. Selenium fed**	273.1	306.7	-33.6	30.23
**Cmpt vs. Trained**	273.1	364.8	-91.71	31.09
**Selenium fed vs. Trained**	306.7	364.8	-58.11	31.79

Soleus Twitch Twitch/Tetanus

Bonferroni's multiple comparisons test	Mean Diff.	95% CI of diff.	Significant?	Summary
Young Control vs. Aged Control	-0.02322	-0.1504 to 0.1040	No	ns
Young Control vs. Cmpt	0.04812	-0.1060 to 0.2022	No	ns
Young Control vs. Selenium fed	-0.01655	-0.1473 to 0.1142	No	ns
Young Control vs. Trained	-0.001638	-0.1661 to 0.1629	No	ns
Aged Control vs. Cmpt	0.07134	-0.08891 to 0.231[illegible]	No	ns
Aged Control vs. Selenium fed	0.006674	-0.1313 to 0.1446	No	ns
Aged Control vs. Trained	0.02158	-0.1487 to 0.1919	No	ns
Cmpt vs. Selenium fed	-0.06467	-0.2277 to 0.0983[illegible]	No	ns
Cmpt vs. Trained	-0.04976	-0.2410 to 0.1414	No	ns
Selenium fed vs. Trained	0.01491	-0.1580 to 0.1879	No	ns

Test details	Mean 1	Mean 2	Mean Diff.	SE of diff.
Young Control vs. Aged Control	0.1964	0.2196	-0.02322	0.04288
Young Control vs. Cmpt	0.1964	0.1482	0.04812	0.05193
Young Control vs. Selenium fed	0.1964	0.2129	-0.01655	0.04406
Young Control vs. Trained	0.1964	0.198	-0.001638	0.05545
Aged Control vs. Cmpt	0.2196	0.1482	0.07134	0.05401
Aged Control vs. Selenium fed	0.2196	0.2129	0.006674	0.0465
Aged Control vs. Trained	0.2196	0.198	0.02158	0.0574
Cmpt vs. Selenium fed	0.1482	0.2129	-0.06467	0.05496
Cmpt vs. Trained	0.1482	0.198	-0.04976	0.06444
Selenium fed vs. Trained	0.2129	0.198	0.01491	0.05829

Soleus Twitch CSA (mm2)

Bonferroni's multiple comparisons test	Mean Diff.	95% CI of diff.	Significant?	Summary
Young Control vs. Aged Control	-0.05202	-0.4557 to 0.3517	No	ns
Young Control vs. Cmpt	-0.03547	-0.4732 to 0.4022	No	ns
Young Control vs. Selenium fed	-0.003122	-0.4529 to 0.4466	No	ns
Young Control vs. Trained	0.08671	-0.3774 to 0.5508	No	ns
Aged Control vs. Cmpt	0.01655	-0.4147 to 0.4478	No	ns
Aged Control vs. Selenium fed	0.0489	-0.3946 to 0.4924	No	ns
Aged Control vs. Trained	0.1387	-0.3193 to 0.5968	No	ns
Cmpt vs. Selenium fed	0.03235	-0.4423 to 0.5070	No	ns
Cmpt vs. Trained	0.1222	-0.3661 to 0.6104	No	ns
Selenium fed vs. Trained	0.08983	-0.4093 to 0.5889	No	ns

Test details	Mean 1	Mean 2	Mean Diff.	SE of diff.
Young Control vs. Aged Control	1.014	1.066	-0.05202	0.1379
Young Control vs. Cmpt	1.014	1.05	-0.03547	0.1495
Young Control vs. Selenium fed	1.014	1.018	-0.003122	0.1537
Young Control vs. Trained	1.014	0.9278	0.08671	0.1586
Aged Control vs. Cmpt	1.066	1.05	0.01655	0.1473
Aged Control vs. Selenium fed	1.066	1.018	0.0489	0.1515
Aged Control vs. Trained	1.066	0.9278	0.1387	0.1565
Cmpt vs. Selenium fed	1.05	1.018	0.03235	0.1622
Cmpt vs. Trained	1.05	0.9278	0.1222	0.1668
Selenium fed vs. Trained	1.018	0.9278	0.08983	0.1705

Soleus Tetanus Peak force (mN/mm2)

Bonferroni's multiple comparisons test	Mean Diff.	95% CI of diff.	Significant?	Summary
Young Control vs. Aged Control	1.147	-4.239 to 6.532	No	ns
Young Control vs. Cmpt	0.2928	-5.774 to 6.360	No	ns
Young Control vs. Selenium fed	1.299	-4.369 to 6.967	No	ns
Young Control vs. Trained	0.4419	-5.407 to 6.291	No	ns
Aged Control vs. Cmpt	-0.8539	-7.102 to 5.394	No	ns
Aged Control vs. Selenium fed	0.1528	-5.709 to 6.014	No	ns
Aged Control vs. Trained	-0.7048	-6.741 to 5.332	No	ns
Cmpt vs. Selenium fed	1.007	-5.487 to 7.500	No	ns
Cmpt vs. Trained	0.1491	-6.503 to 6.801	No	ns
Selenium fed vs. Trained	-0.8575	-7.147 to 5.432	No	ns

Test details	Mean 1	Mean 2	Mean Diff.	SE of diff.
Young Control vs. Aged Control	11.36	10.21	1.147	1.83
Young Control vs. Cmpt	11.36	11.07	0.2928	2.062
Young Control vs. Selenium fed	11.36	10.06	1.299	1.926
Young Control vs. Trained	11.36	10.92	0.4419	1.988
Aged Control vs. Cmpt	10.21	11.07	-0.8539	2.123
Aged Control vs. Selenium fed	10.21	10.06	0.1528	1.992
Aged Control vs. Trained	10.21	10.92	-0.7048	2.051
Cmpt vs. Selenium fed	11.07	10.06	1.007	2.207
Cmpt vs. Trained	11.07	10.92	0.1491	2.261
Selenium fed vs. Trained	10.06	10.92	-0.8575	2.138

Soleus Tetanus TTP (ms)

Bonferroni's multiple comparisons test	Mean Diff.	95% CI of diff.	Significant?	Summary
Young Control vs. Aged Control	-10.07	-24.84 to 4.696	Yes	**
Young Control vs. Cmpt	-14.14	-30.15 to 1.867	No	ns
Young Control vs. Selenium fed	-15.37	-31.82 to 1.086	No	ns
Young Control vs. Trained	-20.72	-37.70 to -3.742	Yes	**
Aged Control vs. Cmpt	-4.074	-19.85 to 11.70	No	ns
Aged Control vs. Selenium fed	-5.297	-21.52 to 10.93	No	ns
Aged Control vs. Trained	-10.65	-27.40 to 6.105	No	ns
Cmpt vs. Selenium fed	-1.223	-18.58 to 16.14	No	ns
Cmpt vs. Trained	-6.575	-24.44 to 11.28	No	ns
Selenium fed vs. Trained	-5.352	-23.61 to 12.90	No	ns

Test details	Mean 1	Mean 2	Mean Diff.	SE of diff.
Young Control vs. Aged Control	505.9	516	-10.07	5.045
Young Control vs. Cmpt	505.9	520	-14.14	5.47
Young Control vs. Selenium fed	505.9	521.3	-15.37	5.621
Young Control vs. Trained	505.9	526.6	-20.72	5.8
Aged Control vs. Cmpt	516	520	-4.074	5.389
Aged Control vs. Selenium fed	516	521.3	-5.297	5.542
Aged Control vs. Trained	516	526.6	-10.65	5.724
Cmpt vs. Selenium fed	520	521.3	-1.223	5.932
Cmpt vs. Trained	520	526.6	-6.575	6.102
Selenium fed vs. Trained	521.3	526.6	-5.352	6.238

Soleus Tetanus HRT (ms)				
**Bonferroni's multiple comparisons test**	**Mean Diff.**	**95% CI of diff.**	**Significant?**	**Summary**
**Young Control vs. Aged Control**	-34.71	-54.16 to -15.26	Yes	****
**Young Control vs. Cmpt**	-17.96	-39.05 to 3.124	No	ns
**Young Control vs. Selenium fed**	-58.24	-79.91 to -36.57	Yes	****
**Young Control vs. Trained**	-27.93	-50.29 to -5.573	Yes	**
**Aged Control vs. Cmpt**	16.74	-4.030 to 37.52	No	ns
**Aged Control vs. Selenium fed**	-23.53	-44.90 to -2.168	Yes	*
**Aged Control vs. Trained**	6.774	-15.29 to 28.84	No	ns
**Cmpt vs. Selenium fed**	-40.28	-63.15 to -17.41	Yes	****
**Cmpt vs. Trained**	-9.971	-33.49 to 13.55	No	ns
**Selenium fed vs. Trained**	30.31	6.261 to 54.35	Yes	**
**Test details**	**Mean 1**	**Mean 2**	**Mean Diff.**	**SE of diff.**
**Young Control vs. Aged Control**	91.49	126.2	-34.71	6.645
**Young Control vs. Cmpt**	91.49	109.5	-17.96	7.204
**Young Control vs. Selenium fed**	91.49	149.7	-58.24	7.403
**Young Control vs. Trained**	91.49	119.4	-27.93	7.639
**Aged Control vs. Cmpt**	126.2	109.5	16.74	7.098
**Aged Control vs. Selenium fed**	126.2	149.7	-23.53	7.3
**Aged Control vs. Trained**	126.2	119.4	6.774	7.539
**Cmpt vs. Selenium fed**	109.5	149.7	-40.28	7.812
**Cmpt vs. Trained**	109.5	119.4	-9.971	8.037
**Selenium fed vs. Trained**	149.7	119.4	30.31	8.215

Soleus Tetanus Duration (ms)				
**Bonferroni's multiple comparisons test**	**Mean Diff.**	**95% CI of diff.**	**Significant?**	**Summary**
**Young Control vs. Aged Control**	-119.3	-201.4 to -37.15	Yes	***
**Young Control vs. Cmpt**	-95.02	-184.1 to -5.968	Yes	*
**Young Control vs. Selenium fed**	-161	-252.5 to -69.50	Yes	****
**Young Control vs. Trained**	-79.2	-173.6 to 15.23	No	ns
**Aged Control vs. Cmpt**	24.27	-63.47 to 112.0	No	ns
**Aged Control vs. Selenium fed**	-41.72	-131.9 to 48.51	No	ns
**Aged Control vs. Trained**	40.09	-53.10 to 133.3	No	ns
**Cmpt vs. Selenium fed**	-65.99	-162.6 to 30.58	No	ns
**Cmpt vs. Trained**	15.82	-83.52 to 115.2	No	ns
**Selenium fed vs. Trained**	81.81	-19.74 to 183.4	No	ns
**Test details**	**Mean 1**	**Mean 2**	**Mean Diff.**	**SE of diff.**
**Young Control vs. Aged Control**	701.6	820.9	-119.3	28.06
**Young Control vs. Cmpt**	701.6	796.6	-95.02	30.42
**Young Control vs. Selenium fed**	701.6	862.6	-161	31.26
**Young Control vs. Trained**	701.6	780.8	-79.2	32.26
**Aged Control vs. Cmpt**	820.9	796.6	24.27	29.98
**Aged Control vs. Selenium fed**	820.9	862.6	-41.72	30.83
**Aged Control vs. Trained**	820.9	780.8	40.09	31.84
**Cmpt vs. Selenium fed**	796.6	862.6	-65.99	32.99
**Cmpt vs. Trained**	796.6	780.8	15.82	33.94
**Selenium fed vs. Trained**	862.6	780.8	81.81	34.7

**Soleus Tetanus 50th Fatigue**				
**Bonferroni's multiple comparisons test**	**Mean Diff.**	**95% CI of diff.**	**Significant?**	**Summary**
**Young Control vs. Aged Control**	0.1071	-0.006194 to 0.2203	Yes	*
**Young Control vs. Cmpt**	-0.0139	-0.1366 to 0.1088	No	ns
**Young Control vs. Selenium fed**	0.1264	0.007417 to 0.2454	Yes	**
**Young Control vs. Trained**	0.03835	-0.08432 to 0.1610	No	ns
**Aged Control vs. Cmpt**	-0.121	-0.2457 to 0.003786	Yes	**
**Aged Control vs. Selenium fed**	0.01935	-0.1018 to 0.1405	No	ns
**Aged Control vs. Trained**	-0.0687	-0.1934 to 0.05604	Yes	***
**Cmpt vs. Selenium fed**	0.1403	0.01033 to 0.2703	Yes	*
**Cmpt vs. Trained**	0.05226	-0.08110 to 0.1856	No	ns
**Selenium fed vs. Trained**	-0.08805	-0.2180 to 0.04192	No	ns
**Test details**	**Mean 1**	**Mean 2**	**Mean Diff.**	**SE of diff.**
**Young Control vs. Aged Control**	0.3349	0.2279	0.1071	0.03848
**Young Control vs. Cmpt**	0.3349	0.3488	-0.0139	0.04169
**Young Control vs. Selenium fed**	0.3349	0.2085	0.1264	0.04044
**Young Control vs. Trained**	0.3349	0.2966	0.03835	0.04169
**Aged Control vs. Cmpt**	0.2279	0.3488	-0.121	0.04239
**Aged Control vs. Selenium fed**	0.2279	0.2085	0.01935	0.04116
**Aged Control vs. Trained**	0.2279	0.2966	-0.0687	0.04239
**Cmpt vs. Selenium fed**	0.3488	0.2085	0.1403	0.04417
**Cmpt vs. Trained**	0.3488	0.2966	0.05226	0.04532
**Selenium fed vs. Trained**	0.2085	0.2966	-0.08805	0.04417

**Soleus Tetanus 100th Fatigue**				
**Bonferroni's multiple comparisons test**	**Mean Diff.**	**95% CI of diff.**	**Significant?**	**Summary**
**Young Control vs. Aged Control**	0.1394	0.003062 to 0.2757	Yes	*
**Young Control vs. Cmpt**	-0.05448	-0.2021 to 0.09316	No	ns
**Young Control vs. Selenium fed**	0.1802	0.03700 to 0.3234	Yes	**
**Young Control vs. Trained**	0.1003	-0.04733 to 0.2479	No	ns
**Aged Control vs. Cmpt**	-0.1938	-0.3440 to -0.04370	Yes	***
**Aged Control vs. Selenium fed**	0.04085	-0.1049 to 0.1866	No	ns
**Aged Control vs. Trained**	-0.03906	-0.1892 to 0.1111	No	ns
**Cmpt vs. Selenium fed**	0.2347	0.07825 to 0.3911	Yes	***
**Cmpt vs. Trained**	0.1548	-0.005717 to 0.3153	No	ns
**Selenium fed vs. Trained**	-0.07991	-0.2363 to 0.07653	No	ns
**Test details**	**Mean 1**	**Mean 2**	**Mean Diff.**	**SE of diff.**
**Young Control vs. Aged Control**	0.5463	0.4069	0.1394	0.04632
**Young Control vs. Cmpt**	0.5463	0.6007	-0.05448	0.05017
**Young Control vs. Selenium fed**	0.5463	0.366	0.1802	0.04867
**Young Control vs. Trained**	0.5463	0.446	0.1003	0.05017
**Aged Control vs. Cmpt**	0.4069	0.6007	-0.1938	0.05102
**Aged Control vs. Selenium fed**	0.4069	0.366	0.04085	0.04954
**Aged Control vs. Trained**	0.4069	0.446	-0.03906	0.05102
**Cmpt vs. Selenium fed**	0.6007	0.366	0.2347	0.05316
**Cmpt vs. Trained**	0.6007	0.446	0.1548	0.05454
**Selenium fed vs. Trained**	0.366	0.446	-0.07991	0.05316

Soleus Tetanus 150th Fatigue				
**Bonferroni's multiple comparisons test**	**Mean Diff.**	**95% CI of diff.**	**Significant?**	**Summary**
**Young Control vs. Aged Control**	0.1451	0.0003160 to 0.2899	**Yes**	*
**Young Control vs. Cmpt**	-0.08987	-0.2463 to 0.06654	No	ns
**Young Control vs. Selenium fed**	0.1481	-0.003794 to 0.3000	No	ns
**Young Control vs. Trained**	0.1239	-0.03256 to 0.2803	No	ns
**Aged Control vs. Cmpt**	-0.235	-0.3914 to -0.07858	**Yes**	***
**Aged Control vs. Selenium fed**	0.002958	-0.1489 to 0.1548	No	ns
**Aged Control vs. Trained**	-0.02127	-0.1777 to 0.1351	No	ns
**Cmpt vs. Selenium fed**	0.2379	0.07497 to 0.4009	**Yes**	***
**Cmpt vs. Trained**	0.2137	0.04651 to 0.3809	**Yes**	**
**Selenium fed vs. Trained**	-0.02423	-0.1872 to 0.1387	No	ns
**Test details**	**Mean 1**	**Mean 2**	**Mean Diff.**	**SE of diff.**
**Young Control vs. Aged Control**	0.633	0.4879	0.1451	0.04916
**Young Control vs. Cmpt**	0.633	0.7229	-0.08987	0.0531
**Young Control vs. Selenium fed**	0.633	0.4849	0.1481	0.05156
**Young Control vs. Trained**	0.633	0.5092	0.1239	0.0531
**Aged Control vs. Cmpt**	0.4879	0.7229	-0.235	0.0531
**Aged Control vs. Selenium fed**	0.4879	0.4849	0.002958	0.05156
**Aged Control vs. Trained**	0.4879	0.5092	-0.02127	0.0531
**Cmpt vs. Selenium fed**	0.7229	0.4849	0.2379	0.05533
**Cmpt vs. Trained**	0.7229	0.5092	0.2137	0.05676
**Selenium fed vs. Trained**	0.4849	0.5092	-0.02423	0.05533

Statistics of data from Table 3.

Resting [Ca2+] (nM)				
**Bonferroni's multiple comparisons test**	**Mean Diff.**	**95% CI of diff.**	**Significant?**	**Summary**
**Young Control vs. Aged Control**	-6.636	-12.28 to -0.9904	Yes	***
**Young Control vs. Cmpt**	-16.83	-24.56 to -9.114	Yes	****
**Young Control vs. Selenium fed**	3.406	-3.594 to 10.41	No	ns
**Young Control vs. Trained**	1.448	-7.928 to 10.82	No	ns
**Aged Control vs. Cmpt**	-10.2	-18.64 to -1.754	Yes	**
**Aged Control vs. Selenium fed**	10.04	2.252 to 17.83	Yes	***
**Aged Control vs. Trained**	8.084	-1.896 to 18.06	Yes	*
**Cmpt vs. Selenium fed**	20.24	10.84 to 29.65	Yes	****
**Cmpt vs. Trained**	18.28	6.998 to 29.57	Yes	****
**Selenium fed vs. Trained**	-1.959	-12.76 to 8.846	No	ns
**Test details**	**Mean 1**	**Mean 2**	**Mean Diff.**	**SE of diff.**
**Young Control vs. Aged Control**	71.47	78.11	-6.636	1.969
**Young Control vs. Cmpt**	71.47	88.31	-16.83	2.693
**Young Control vs. Selenium fed**	71.47	68.06	3.406	2.442
**Young Control vs. Trained**	71.47	70.02	1.448	3.27
**Aged Control vs. Cmpt**	78.11	88.31	-10.2	2.946
**Aged Control vs. Selenium fed**	78.11	68.06	10.04	2.718
**Aged Control vs. Trained**	78.11	70.02	8.084	3.481
**Cmpt vs. Selenium fed**	88.31	68.06	20.24	3.28
**Cmpt vs. Trained**	88.31	70.02	18.28	3.936
**Selenium fed vs. Trained**	68.06	70.02	-1.959	3.769
**Δ [Ca2+] (μM)**				
**Bonferroni's multiple comparisons test**	**Mean Diff.**	**95% CI of diff.**	**Significant?**	**Summary**
**Young Control vs. Aged Control**	77.38	-244.3 to 399.1	No	ns
**Young Control vs. Cmpt**	480.4	51.68 to 909.0	Yes	*
**Young Control vs. Selenium fed**	-436.6	-851.1 to -22.08	Yes	*
**Young Control vs. Trained**	-1069	-1556 to -581.7	Yes	****
**Aged Control vs. Cmpt**	403	-51.35 to 857.3	Yes	***
**Aged Control vs. Selenium fed**	-514	-954.9 to -73.00	Yes	***
**Aged Control vs. Trained**	-1146	-1656 to -636.4	Yes	****
**Cmpt vs. Selenium fed**	-916.9	-1441 to -392.8	Yes	****
**Cmpt vs. Trained**	-1549	-2133 to -965.9	Yes	****
**Selenium fed vs. Trained**	-632.4	-1206 to -59.31	Yes	*
**Test details**	**Mean 1**	**Mean 2**	**Mean Diff.**	**SE of diff.**
**Young Control vs. Aged Control**	643.7	566.3	77.38	111.8
**Young Control vs. Cmpt**	643.7	163.4	480.4	149
**Young Control vs. Selenium fed**	643.7	1080	-436.6	144
**Young Control vs. Trained**	643.7	1713	-1069	169.3
**Aged Control vs. Cmpt**	566.3	163.4	403	157.9
**Aged Control vs. Selenium fed**	566.3	1080	-514	153.2
**Aged Control vs. Trained**	566.3	1713	-1146	177.2
**Cmpt vs. Selenium fed**	163.4	1080	-916.9	182.1
**Cmpt vs. Trained**	163.4	1713	-1549	202.7
**Selenium fed vs. Trained**	1080	1713	-632.4	199.1

Ca2+ Release Flux (µM/s)

Bonferroni's multiple comparisons test	Mean Diff.	95% CI of diff.	Significant?	Summary
Young Control vs. Aged Control	163.6	0.3461 to 326.8	Yes	**
Young Control vs. Cmpt	458	241.5 to 674.5	Yes	****
Young Control vs. Selenium fed	-155.8	-365.2 to 53.61	No	ns
Young Control vs. Trained	-471.8	-717.6 to -226.0	Yes	****
Aged Control vs. Cmpt	294.4	66.31 to 522.5	Yes	***
Aged Control vs. Selenium fed	-319.4	-540.8 to -97.98	Yes	***
Aged Control vs. Trained	-635.4	-891.4 to -379.3	Yes	****
Cmpt vs. Selenium fed	-613.8	-876.9 to -350.6	Yes	****
Cmpt vs. Trained	-929.8	-1223 to -636.8	Yes	****
Selenium fed vs. Trained	-316	-603.7 to -28.23	Yes	*

Test details	Mean 1	Mean 2	Mean Diff.	SE of diff.
Young Control vs. Aged Control	644.7	481.2	163.6	56.68
Young Control vs. Cmpt	644.7	186.8	458	75.19
Young Control vs. Selenium fed	644.7	800.5	-155.8	72.73
Young Control vs. Trained	644.7	1117	-471.8	85.36
Aged Control vs. Cmpt	481.2	186.8	294.4	79.21
Aged Control vs. Selenium fed	481.2	800.5	-319.4	76.89
Aged Control vs. Trained	481.2	1117	-635.4	88.93
Cmpt vs. Selenium fed	186.8	800.5	-613.8	91.39
Cmpt vs. Trained	186.8	1117	-929.8	101.7
Selenium fed vs. Trained	800.5	1117	-316	99.93

Ca2+ Amount Released (µM)

Bonferroni's multiple comparisons test	Mean Diff.	95% CI of diff.	Significant?	Summary
Young Control vs. Aged Control	5.599	-404.2 to 415.4	No	ns
Young Control vs. Cmpt	905.5	363.2 to 1448	Yes	****
Young Control vs. Selenium fed	-23.43	-548.1 to 501.2	No	ns
Young Control vs. Trained	-384.3	-999.5 to 230.9	No	ns
Aged Control vs. Cmpt	899.9	330.4 to 1469	Yes	***
Aged Control vs. Selenium fed	-29.03	-581.8 to 523.7	No	ns
Aged Control vs. Trained	-389.9	-1029 to 249.4	Yes	**
Cmpt vs. Selenium fed	-928.9	-1586 to -271.9	Yes	**
Cmpt vs. Trained	-1290	-2021 to -558.4	Yes	****
Selenium fed vs. Trained	-360.9	-1079 to 357.5	No	ns

Test details	Mean 1	Mean 2	Mean Diff.	SE of diff.
Young Control vs. Aged Control	1332	1327	5.599	142.3
Young Control vs. Cmpt	1332	426.7	905.5	188.2
Young Control vs. Selenium fed	1332	1356	-23.43	182.1
Young Control vs. Trained	1332	1716	-384.3	213.6
Aged Control vs. Cmpt	1327	426.7	899.9	197.7
Aged Control vs. Selenium fed	1327	1356	-29.03	191.9
Aged Control vs. Trained	1327	1716	-389.9	222
Cmpt vs. Selenium fed	426.7	1356	-928.9	228.1
Cmpt vs. Trained	426.7	1716	-1290	253.9
Selenium fed vs. Trained	1356	1716	-360.9	249.4

Statistics of data from Figure 5.

Full Length RyR1 (relative intensity)

Bonferroni's multiple comparisons test	Mean Diff.	95% CI of diff.	Significant?	Summary
Young Control vs. Aged Control	5.548	3.690 to 7.406	Yes	****
Young Control vs. Cmpt	5.807	3.948 to 7.665	Yes	****
Young Control vs. Selenium fed	5.018	3.160 to 6.876	Yes	****
Young Control vs. Trained	-0.03095	-1.889 to 1.827	No	ns
Aged Control vs. Cmpt	0.2584	-1.403 to 1.920	No	ns
Aged Control vs. Selenium fed	-0.5298	-2.192 to 1.132	No	ns
Aged Control vs. Trained	-5.579	-7.241 to -3.917	Yes	****
Cmpt vs. Selenium fed	-0.7882	-2.450 to 0.8737	No	ns
Cmpt vs. Trained	-5.837	-7.499 to -4.176	Yes	****
Selenium fed vs. Trained	-5.049	-6.711 to -3.387	Yes	****

Test details	Mean 1	Mean 2	Mean Diff.	SE of diff.
Young Control vs. Aged Control	6.548	1	5.548	0.5986
Young Control vs. Cmpt	6.548	0.7416	5.807	0.5986
Young Control vs. Selenium fed	6.548	1.53	5.018	0.5986
Young Control vs. Trained	6.548	6.579	-0.03095	0.5986
Aged Control vs. Cmpt	1	0.7416	0.2584	0.5354
Aged Control vs. Selenium fed	1	1.53	-0.5298	0.5354
Aged Control vs. Trained	1	6.579	-5.579	0.5354
Cmpt vs. Selenium fed	0.7416	1.53	-0.7882	0.5354
Cmpt vs. Trained	0.7416	6.579	-5.837	0.5354
Selenium fed vs. Trained	1.53	6.579	-5.049	0.5354

Relative RyR1 Degradation

Bonferroni's multiple comparisons test	Mean Diff.	95% CI of diff.	Significant?	Summary
Young Control vs. Aged Control	-0.4585	-0.7529 to -0.1640	Yes	**
Young Control vs. Cmpt	-0.5215	-0.8159 to -0.2270	Yes	***
Young Control vs. Selenium fed	-0.4336	-0.7281 to -0.1392	Yes	**
Young Control vs. Trained	-0.2593	-0.5538 to 0.03516	No	ns
Aged Control vs. Cmpt	-0.06302	-0.3264 to 0.2004	No	ns
Aged Control vs. Selenium fed	0.02483	-0.2386 to 0.2882	No	ns
Aged Control vs. Trained	0.1992	-0.06423 to 0.4625	Yes	*
Cmpt vs. Selenium fed	0.08785	-0.1755 to 0.3512	No	ns
Cmpt vs. Trained	0.2622	-0.001209 to 0.5256	No	ns
Selenium fed vs. Trained	0.1743	-0.08905 to 0.4377	No	ns

Test details	Mean 1	Mean 2	Mean Diff.	SE of diff.
Young Control vs. Aged Control	0.1603	0.6188	-0.4585	0.09487
Young Control vs. Cmpt	0.1603	0.6818	-0.5215	0.09487
Young Control vs. Selenium fed	0.1603	0.5939	-0.4336	0.09487
Young Control vs. Trained	0.1603	0.4196	-0.2593	0.09487
Aged Control vs. Cmpt	0.6188	0.6818	-0.06302	0.08485
Aged Control vs. Selenium fed	0.6188	0.5939	0.02483	0.08485
Aged Control vs. Trained	0.6188	0.4196	0.1992	0.08485
Cmpt vs. Selenium fed	0.6818	0.5939	0.08785	0.08485
Cmpt vs. Trained	0.6818	0.4196	0.2622	0.08485
Selenium fed vs. Trained	0.5939	0.4196	0.1743	0.08485

Statistics of data from Figure 6.

SERCA1 Relative Intensity				
**Bonferroni's multiple comparisons test**	**Mean Diff.**	**95% CI of diff.**	**Significant?**	**Summary**
**Control vs. Cmpt**	0.08006	-1.453 to 1.613	No	ns
**Control vs. Selenium**	-0.03594	-1.569 to 1.497	No	ns
**Control vs. Trained**	-0.1249	-1.658 to 1.408	No	ns
**Cmpt vs. Selenium**	-0.116	-1.649 to 1.417	No	ns
**Cmpt vs. Trained**	-0.205	-1.738 to 1.328	No	ns
**Selenium vs. Trained**	-0.08899	-1.622 to 1.444	No	ns
**Test details**	**Mean 1**	**Mean 2**	**Mean Diff.**	**SE of diff.**
**Control vs. Cmpt**	1	0.9199	0.08006	0.4406
**Control vs. Selenium**	1	1.036	-0.03594	0.4406
**Control vs. Trained**	1	1.125	-0.1249	0.4406
**Cmpt vs. Selenium**	0.9199	1.036	-0.116	0.4406
**Cmpt vs. Trained**	0.9199	1.125	-0.205	0.4406
**Selenium vs. Trained**	1.036	1.125	-0.08899	0.4406

DHPR Relative Intensity				
**Bonferroni's multiple comparisons test**	**Mean Diff.**	**95% CI of diff.**	**Significant?**	**Summary**
**Control vs. Cmpt**	-0.1757	-1.266 to 0.9143	No	ns
**Control vs. Selenium**	-0.3757	-1.466 to 0.7144	No	ns
**Control vs. Trained**	-0.1046	-1.195 to 0.9855	No	ns
**Cmpt vs. Selenium**	-0.2	-1.290 to 0.8901	No	ns
**Cmpt vs. Trained**	0.07113	-1.019 to 1.161	No	ns
**Selenium vs. Trained**	0.2711	-0.8189 to 1.361	No	ns
**Test details**	**Mean 1**	**Mean 2**	**Mean Diff.**	**SE of diff.**
**Control vs. Cmpt**	1	1.176	-0.1757	0.3133
**Control vs. Selenium**	1	1.376	-0.3757	0.3133
**Control vs. Trained**	1	1.105	-0.1046	0.3133
**Cmpt vs. Selenium**	1.176	1.376	-0.2	0.3133
**Cmpt vs. Trained**	1.176	1.105	0.07113	0.3133
**Selenium vs. Trained**	1.376	1.105	0.2711	0.3133

Statistics of data from Supplementary Figure 3.

SERCA1 Relative Intensity				
**Bonferroni's multiple comparisons test**	**Mean Diff.**	**95% CI of diff.**	**Significant?**	**Summary**
**Young Control vs. Young Cmpt**	0.1562	-0.4361 to 0.7485	No	ns
**Young Control vs. Aged Control**	-0.09554	-0.7353 to 0.5442	No	ns
**Young Control vs. Aged Cmpt**	-0.05284	-0.6926 to 0.5869	No	ns
**Young Cmpt vs. Aged Control**	-0.2517	-0.8915 to 0.3880	No	ns
**Young Cmpt vs. Aged Cmpt**	-0.209	-0.8488 to 0.4307	No	ns
**Aged Control vs. Aged Cmpt**	0.0427	-0.6412 to 0.7267	No	ns
**Test details**	**Mean 1**	**Mean 2**	**Mean Diff.**	**SE of diff.**
**Young Control vs. Young Cmpt**	1	0.8438	0.1562	0.1808
**Young Control vs. Aged Control**	1	1.096	-0.09554	0.1952
**Young Control vs. Aged Cmpt**	1	1.053	-0.05284	0.1952
**Young Cmpt vs. Aged Control**	0.8438	1.096	-0.2517	0.1952
**Young Cmpt vs. Aged Cmpt**	0.8438	1.053	-0.209	0.1952
**Aged Control vs. Aged Cmpt**	1.096	1.053	0.0427	0.2087

DHPR Relative Intensity				
**Bonferroni's multiple comparisons test**	**Mean Diff.**	**95% CI of diff.**	**Significant?**	**Summary**
**Young Control vs. Young Cmpt**	0.1233	-0.7339 to 0.9805	No	ns
**Young Control vs. Aged Control**	0.347	-0.3871 to 1.081	No	ns
**Young Control vs. Aged Cmpt**	0.2007	-0.5819 to 0.9832	No	ns
**Young Cmpt vs. Aged Control**	0.2237	-0.6616 to 1.109	No	ns
**Young Cmpt vs. Aged Cmpt**	0.07738	-0.8485 to 1.003	No	ns
**Aged Control vs. Aged Cmpt**	-0.1463	-0.9595 to 0.6669	No	ns
**Test details**	**Mean 1**	**Mean 2**	**Mean Diff.**	**SE of diff.**
**Young Control vs. Young Cmpt**	1	0.8767	0.1233	0.2793
**Young Control vs. Aged Control**	1	0.653	0.347	0.2392
**Young Control vs. Aged Cmpt**	1	0.7993	0.2007	0.255
**Young Cmpt vs. Aged Control**	0.8767	0.653	0.2237	0.2885
**Young Cmpt vs. Aged Cmpt**	0.8767	0.7993	0.07738	0.3017
**Aged Control vs. Aged Cmpt**	0.653	0.7993	-0.1463	0.265

Statistics of data from Figure 7.

Sepn Relative Intensity				
**Bonferroni's multiple comparisons test**	**Mean Diff.**	**95% CI of diff.**	**Significant?**	**Summary**
**Neonatal vs. Young**	0.07405	-0.3268 to 0.4749	No	ns
**Neonatal vs. Aged**	0.129	-0.2718 to 0.5298	Yes	*
**Neonatal vs. Selenium**	-0.517	-0.9178 to -0.1162	Yes	**
**Young vs. Aged**	0.05495	-0.3459 to 0.4558	No	ns
**Young vs. Selenium**	-0.5911	-0.9919 to -0.1902	Yes	**
**Aged vs. Selenium**	-0.646	-1.047 to -0.2452	Yes	**
**Test details**	**Mean 1**	**Mean 2**	**Mean Diff.**	**SE of diff.**
**Neonatal vs. Young**	0.1866	0.1125	0.07405	0.1271
**Neonatal vs. Aged**	0.1866	0.05756	0.129	0.1271
**Neonatal vs. Selenium**	0.1866	0.7036	-0.517	0.1271
**Young vs. Aged**	0.1125	0.05756	0.05495	0.1271
**Young vs. Selenium**	0.1125	0.7036	-0.5911	0.1271
**Aged vs. Selenium**	0.05756	0.7036	-0.646	0.1271
